# Hypoxia and heat stress affect epithelial integrity in a Caco-2/HT-29 co-culture

**DOI:** 10.1038/s41598-021-92574-5

**Published:** 2021-06-23

**Authors:** Puqiao Lian, Saskia Braber, Soheil Varasteh, Harry J. Wichers, Gert Folkerts

**Affiliations:** 1 Division of Pharmacology, Department of Pharmaceutical Sciences, Faculty of Science, Utrecht Institute for Pharmaceutical Sciences, Utrecht University, Universiteitsweg 99, 3584 CG Utrecht, The Netherlands; 2 Food and Biobased Research, Wageningen University and Research, Wageningen, The Netherlands

**Keywords:** Physiology, Gastroenterology, Pathogenesis

## Abstract

Hypoxia and hyperthermia, which can be induced by high environmental temperature or strenuous exercise, are two common stressors that affect intestinal epithelial integrity and lead to multiple clinical symptoms. In this study, we developed an *in-vitro* intestinal monolayer model using two human colonic epithelial cell lines, Caco-2 and HT-29, co-cultured in Transwell inserts, and investigated the effects of heat treatment and/or hypoxia on the epithelial barrier function. The monolayer with a ratio of 9:1 (Caco-2:HT-29) showed high trans-epithelial electrical resistance (TEER), low Lucifer Yellow permeability and high mucin production. Hyperthermia and/or hypoxia exposure (2 h) triggered heat shock and oxidative stress responses. HSP-70 and HSF-1 protein levels were up-regulated by hyperthermia, which were further enhanced when hyperthermia was combined with hypoxia. Increased HIF-1α protein expression and Nrf2 nuclear translocation was only caused by hypoxia. Hyperthermia and/or hypoxia exposure disrupted the established monolayer by increasing paracellular permeability, decreasing ZO-1, claudin-3 and occludin protein/mRNA expression, while enhancing E-cadherin protein expression. Tight junction protein distribution in the monolayer was also modulated by the hyperthermia and/or hypoxia exposure. In addition, transcription levels of mucin genes, MUC-2 and MUC-5AC, were increased after 2 h of hyperthermia and/or hypoxia exposure. In conclusion, this Caco-2/HT-29 cell model is valid and effective for studying detrimental effects of hyperthermia and/or hypoxia on intestinal barrier function and related heat shock and oxidative stress pathways and can be used to investigate possible interventions to reverse hyperthermia and/or hypoxia-induced intestinal epithelial injury.

## Introduction

The intestinal epithelium acts as a barrier between the internal and external environments and plays an important role in absorbing nutrient substances into the circulation and limiting the infiltration of harmful substances. The epithelium is composed of a monolayer of absorptive enterocytes and specialized secretory cells, such as goblet cells^1^.

Intestinal epithelial integrity is dependent on the organization of the cell–cell junctional complexes, including tight junctions (TJs), adherens junctions (AJs) and desmosomes^2^. TJ proteins contain claudins and occludins, which interact with each other on their extracellular sides to promote junction assembly, and the zonula occludens (ZO) family, ZO-1, ZO-2 and ZO-3, which provide intracellular structural support^3, 4^. E-cadherin is the most essential cadherin present on the epithelial surface responsible for AJ formation. Its function is to hinge at the neighbour cell by another E-cadherin^5^. Desmosomes are structurally similar to AJs but contain special cadherins that help epithelia in building mechanical strength and signalling^6^.

Enterocytes that make up more than 80% of all intestinal cells highly express TJs/AJs proteins, thus forming a tight epithelial layer^7, 8^. Goblet cells protect the epithelium from the luminal contents by secreting mucins and forming a mucous buffer layer^9^. Both the junctional network and the mucous layer protect the intestinal epithelial integrity. Any factor that adversely affects the expression or localization of intercellular junctions or the mucosal barrier may lead to loss of epithelial integrity, which is possibly involved in gastro-intestinal (GI) disorders^9^.

Recent investigations revealed that the GI tract is susceptible to acute and chronic hypoxia (lack of oxygen)^10, 11^. However, in some conditions, such as high environmental temperature or strenuous exercise, increased body temperature results in increased peripheral circulation and reduced blood flow to the internal organs, including the GI tract. Hypoxia can induce intestinal villous ischemia and bacterial translocation, which further leads to local and systemic inflammation, such as inflammatory bowel disease (IBD) and gut-derived septic complications^12–15^. Heat exposure can cause morphological change of villus height and crypt depth, infiltration of macrophage-like cells into the submucosa and direct epithelial damage^16–18^. When hypoxia occurs simultaneously with a heat stimulus, synergic deleterious effects on gut integrity may occur, however knowledge about this topic is scarce.

Hyperthermia-induced hypoxia in the intestine leads to disturbance of the balance between the production of reactive oxygen species (ROS) and the antioxidant defence system^19, 20^, and loosening of tight junctions, which allows the penetration of luminal toxins, resulting in a further inflammatory response while heat stress (HS) occurs. HS is a stress state manifested by heat shock response and oxidative stress response, which are two resilience pathways in response to heat stress mediated cytotoxicity. As a result of high body temperature, elevated levels of dissociated heat shock proteins (HSPs) induce translocation and trimerization of heat shock factor-1 (HSF1) into the nucleus, where it binds to the regulatory heat shock elements (HSE) in the promoter regions of HSP genes, subsequently initiating the transcription of HSP genes contributing to protect TJs/AJs from aggregation or misfolding^21^. The oxidative stress induced by HS, results in liberation of nuclear factor erythroid 2 related factor 2 (Nrf2) from Kelch-like ECH-associated protein 1 (Keap1) and the following translocation of Nrf2 into the nucleus, where it binds to the antioxidant response element (ARE) in the promotor region of antioxidant target genes, driving oxidative resilience pathways.

The human colorectal epithelium cell line, Caco-2, which expresses characteristics of absorptive enterocytes upon differentiation, is widely used for studies on intestinal barrier function^22–24^. However, Caco-2 cells are mostly reported as non/low-mucus producing cells and do not or hardly express mucin-related genes, such as MUC-2 and MUC-6^25^. Compared to Caco-2, the key characteristic of HT-29, another human colorectal adenocarcinoma cell line, is the relative high expression and release of mucin. Through the combination of these two human colorectal epithelial cell types, Caco-2 cells representing the intestinal absorptive enterocytes and form a tight intestinal epithelial barrier, and HT-29 cells representing mucin-producing cells, the physiological situation in this in vitro model, mimics better the in vivo anatomy and physiology. In this study, we established an in vitro co-culture model of these two cell lines. This is in agreement with other studies using Caco-2 cells in combination with other cell lines (such as HT-29, HT-29-MTX and LS174T) in co-culture models to simulate the human intestinal epithelial layer^26–28^. The co-cultures were established on 2D Transwell inserts to mechanistically investigate the interaction of hypoxia and HS challenges on intestinal epithelium as measured by intestinal barrier function, heat shock response and oxidative stress response.

## Results

### Determination of the optimal combination of Caco-2 and HT-29 cells in a co-culture model

**Figure 1:**
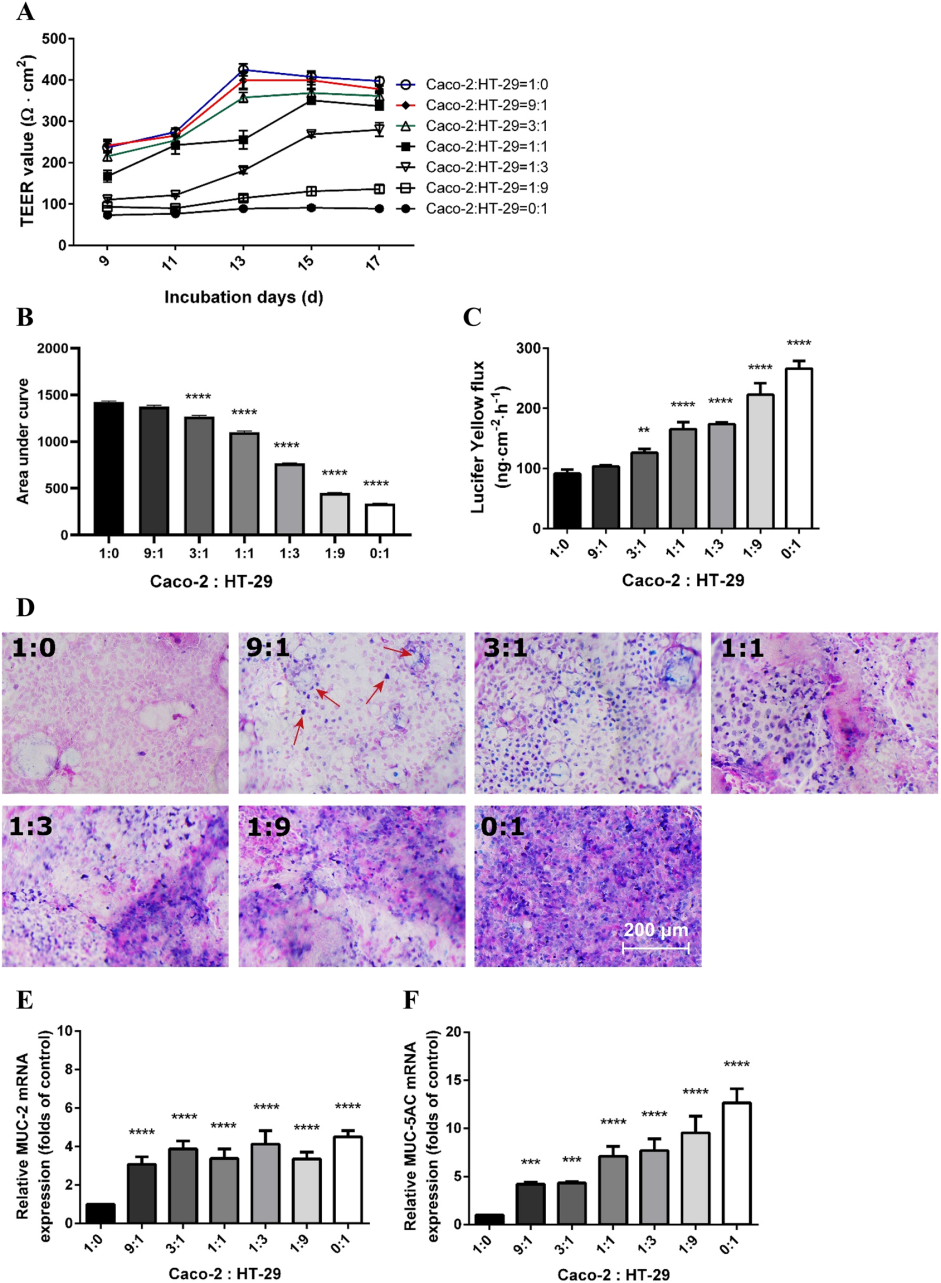
The monolayer integrity and mucus expression in the co-culture model at different ratios of Caco-2 and HT-29 cells. (**A**) TEER values of the cells grown on Transwell membranes. The cell cultures were subjected to TEER evaluation every other day since day 9. (**B**) Areas under the curves of TEER values of the co-culture monolayer calculated for statistical analysis. (**C**) Lucifer Yellow permeability assay of different combinations of Caco-2 and HT-29 cells at day 17. (**D**) Alcian Blue/nuclear fast red staining on Transwell membranes acquired by Nikon Plan 10 × /0.25 objective lens. Pink areas indicated nuclei and blue/purple areas indicated deposition of mucus. Mucin-related mRNA MUC-2 (**E**) and MUC-5AC (**F**) levels assessed by qRT-PCR. The target genes were normalized with reference gene β-actin. All values were presented as means ± SEM (N = 3, n = 3). Statistical differences were analyzed by Two-way analysis of variance (ANOVA), with Bonferroni post-hoc test. **p < 0.01, ***p < 0.001, ****p < 0.0001; significant different from Caco-2:HT-29 = 1:0 group.

A well-established intestinal epithelial co-culture model should have a tight barrier, produce mucus and express mucin-related genes, so the optimal combination of Caco-2 and HT-29 cells was determined by measuring epithelial monolayer integrity and mucus production. The cells were seeded in different combinations and allowed to differentiate. The TEER values increased along with the culture time, being highest at a ratio of Caco-2:HT-29 = 1:0 and lowest in 0:1 group. The TEER values of 100% Caco-2 cells reached 424.7 ± 14.2 Ω × cm^2^ at day 13 and remained at a plateau of around 400 Ω × cm^2^ till the end of the experiment (day 17) (Fig. 1A, blue line). The Caco-2:HT-29 = 9:1 and 3:1 ratios showed similar results; the TEER values also reached a plateau of around 400 Ω × cm^2^ towards the end of the experiment (Fig. 1A, red and green lines). The TEER values of the 1:1 also reached a plateau (350.9 ± 8.3 Ω × cm^2^), but 2 days later than the above mentioned groups. In Caco-2:HT-29 = 1:3, 1:9 and 0:1 ratios, the monolayer could hardly establish epithelial integrity as observed in low TEER values (< 300 Ω × cm^2^). TEER values of individual groups were summarized as areas under the curve (AUC) and this measure was used for statistical analysis (Fig. 1B). At day 17, the cells were subjected to a Lucifer Yellow permeability assay. After 4 h of incubation, the fluorecent flux increased at the basolateral side as the relative proportion of Caco-2 decreased (Fig. 1C). In the 9:1 ratio no change in Lucifer Yellow flux was observed, but in all other ratios a significant increase at the basolateral side was found.

The co-culture was subsequently stained with Alcian Blue/Nuclear Fast Red. The blue area indicates mucus production. No mucus production was observed in the 1:0 ratio group (Fig. 1D). In the 9:1 group, the blue color was scattering around the pink nuclei (Fig. 1D, arrows). As the HT-29 proportion increased, the mucus production (blue staining) increased as well (Fig. 1D). The qRT-PCR results also demonstrated that the transcription of two mucin-related genes, MUC-2 and MUC-5AC, enhanced significantly when HT-29 cells were present (Fig. 1E,F).

From this set of experiments, it can be concluded that the Caco-2:HT-29 = 9:1 ratio showed a tight intestinal barrier and mucus production, and enhanced expression of mucus genes was present. Therefore, this ratio was used in further experiments to investigate the effect of heat stress with or without hypoxia.

### Two hours of heat treatment ± hypoxia disrupts intestinal epithelial integrity in the co-culture model and does not affect cell viability

The cytotoxicity of hypoxia on Caco-2 or HT-29 cells was determined by different exposure times (0 min to 24 h). LDH release started to increase significantly in Caco-2 after 4 h of hypoxia (Figure S1A). In HT-29 cells, LDH release peaked after 2 h of hypoxia exposure, but the difference was not statistically significant (Figure S1B). In addition, the cytotoxic effect of temperature alone or in combination with hypoxia was investigated on the separate sell lines. LDH results showed that 2 h of heat treat stress did not result in additional cytotoxic effects to either Caco-2 and HT-29 cells and the combination of heat treatment + hypoxia for 2 h did not significantly affect the cell viability of both cell lines.(Figure S1C,D). Since 2 h of hypoxia exposure is the maximum non-toxic exposure time for both cell lines, this time point was selected for further experiments.

**Figure 2:**
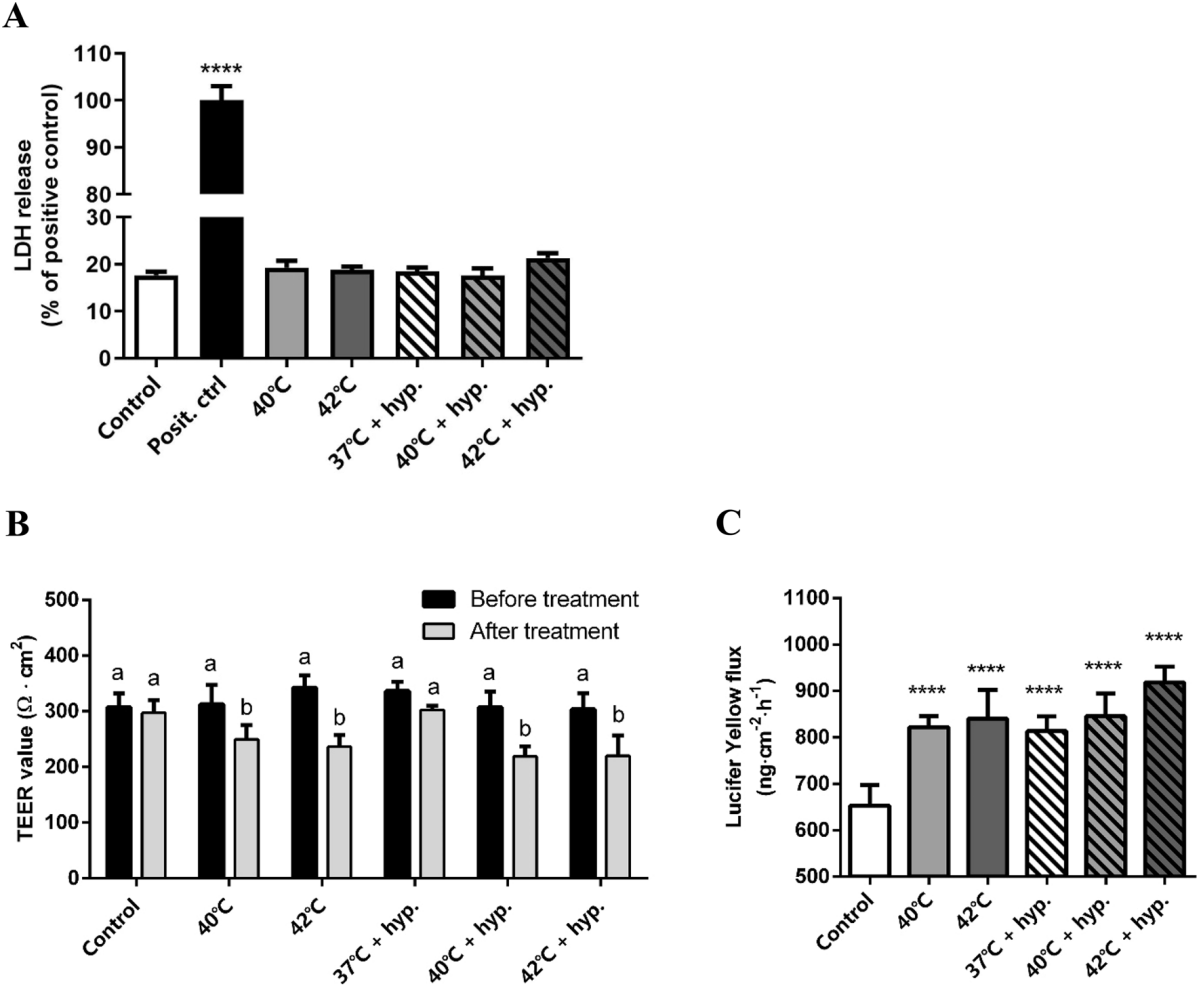
Effects of hypoxia ± heat treatment on cell viability and monolayer integrity in the co-culture of Caco-2 and HT-29 cells. (**A**) LDH release of Caco-2 and HT-29 cells co-culture after 2 h of hypoxia ± heat treatment. In the positive control group, the cells were lysed with lysis buffer. (**B**) TEER values of the co-culture monolayer before and after 2 h of hypoxia ± heat treatment. (**C**) Lucifer Yellow permeability assay of the co-culture monolayer after 2 h of hypoxia ± heat treatment. All values were presented as means ± SEM (N = 3, n = 3). Statistical differences were analyzed by two-way ANOVA followed by the Bonferroni’s multiple comparison test. ****p < 0.0001; significant different from control (**A**, **B** and **D**). Means without a common letter differ at *p* < 0.05 (**C**). *posit. ctrl* positive control; *hyp.* hypoxia.

Next, the cytotoxic effect of high temperature alone or in combination with hypoxia was investigated in the co-culture model. Cell viability remained unaffected by higher temperatures (40 ℃ and 42 ℃) with or without hypoxia for 2 h (Fig. 2A). To investigate the effect of hypoxia and/or heat treatment on epithelial integrity, Caco-2 and HT-29, in a ratio of 9:1 in inserts, were exposed to hypoxia and high temperatures (40 ℃ and 42 ℃) for 2 h. Exposure to 40 ℃ or 42 ℃ reduced TEER values in 2 h in both the hypoxia and the normoxia group (Fig. 2B). In addition, the Lucifer Yellow flux was increased after hypoxia and/or heat treatment (Fig. 2C). Single hypoxia treatment at 37℃ did not affect TEER values, but did increase Lucifer Yellow permeability. This assay was also performed in separate Caco-2 or HT-29 cells (Figure S2). The results showed that HT-29 cells did not form a tight epithelial barrier, as the TEER values were quite low, while Caco-2 cells established a tight epithelial barrier with high resistance. However, after 2-h hypoxia and 42 ℃ treatment, TEER values and Lucifer Yellow permeability did not change significantly in the separate cell lines.

### Heat treatment and/or hypoxia triggers heat shock and oxidative stress responses

**Figure 3:**
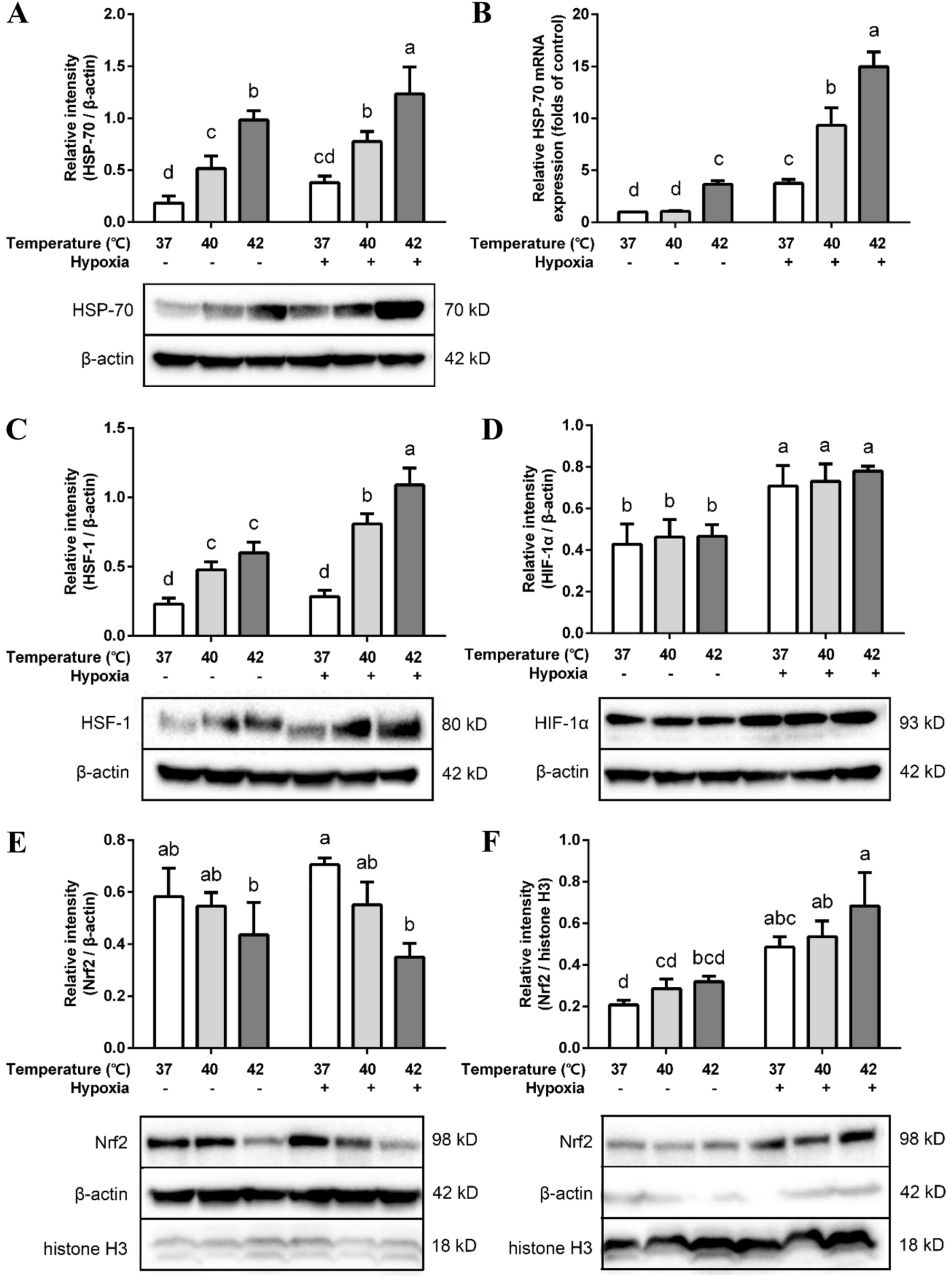
Effects of hypoxia and/or heat treatment on resilience pathways-related proteins in the co-culture model. Relative protein expression of HSP-70 (**A**), HSF-1 (**C**), HIF-1α (**D**) and cytoplasmic (**E**) and nuclear Nrf2 (**F**) assessed by Western Blot. All target proteins were normalized to reference protein β-actin (total/cytoplasmic) or histone H3 (nuclear). (**B**) HSP-70 mRNA levels assessed by qRT-PCR. The target genes were normalized with housekeeping gene β-actin. All values were presented as means ± SD (N = 3, n = 3). Statistical differences were analyzed by two-way ANOVA followed by the Bonferroni’s multiple comparison test. Means without a common letter differ at *p* < 0.05.

Heat treatment increased HSP-70 protein and mRNA expression within 2 h (Fig. 3A,B) and when temperature increased, HSP-70 expression increased as well. Hypoxia alone did not significantly increase HSP-70 protein expression (Fig. 3A), while the HSP70 mRNA expression was higher after hypoxia exposure and in combination with heat treatment the HSP-70 expression was further enhanced (Fig. 3B).

HSF-1 protein expression increased concomitantly with temperature (Fig. 3C). Hypoxia exposure alone did not affect HSF-1 expression, but an additional increase in HSF-1 expression was observed when hypoxia was combined with higher temperatures.

HIF-1α protein expression was not affected by high temperatures, but an increase in protein expression was observed after hypoxia exposure (Fig. 3D). However, there was no significant difference in HIF-1α mRNA expression between hypoxia-treated groups and normoxia groups (Figure S3).

Cytoplasmic and nuclear Nrf2 protein expression was detected in the cell lysate. In the nuclear constituent part, Nrf2 protein expression was enhanced after 2 h of hypoxia exposure (Fig. 3F). In the cytoplasm, Nrf2 protein expression in the hypoxia group remained almost the same as its normoxic counterpart, while in combination with high temperatures the Nrf2 expression was reduced (Fig. 3E).

### Heat treatment and/or hypoxia affects TJs and AJs protein and mRNA expression

**Figure 4:**
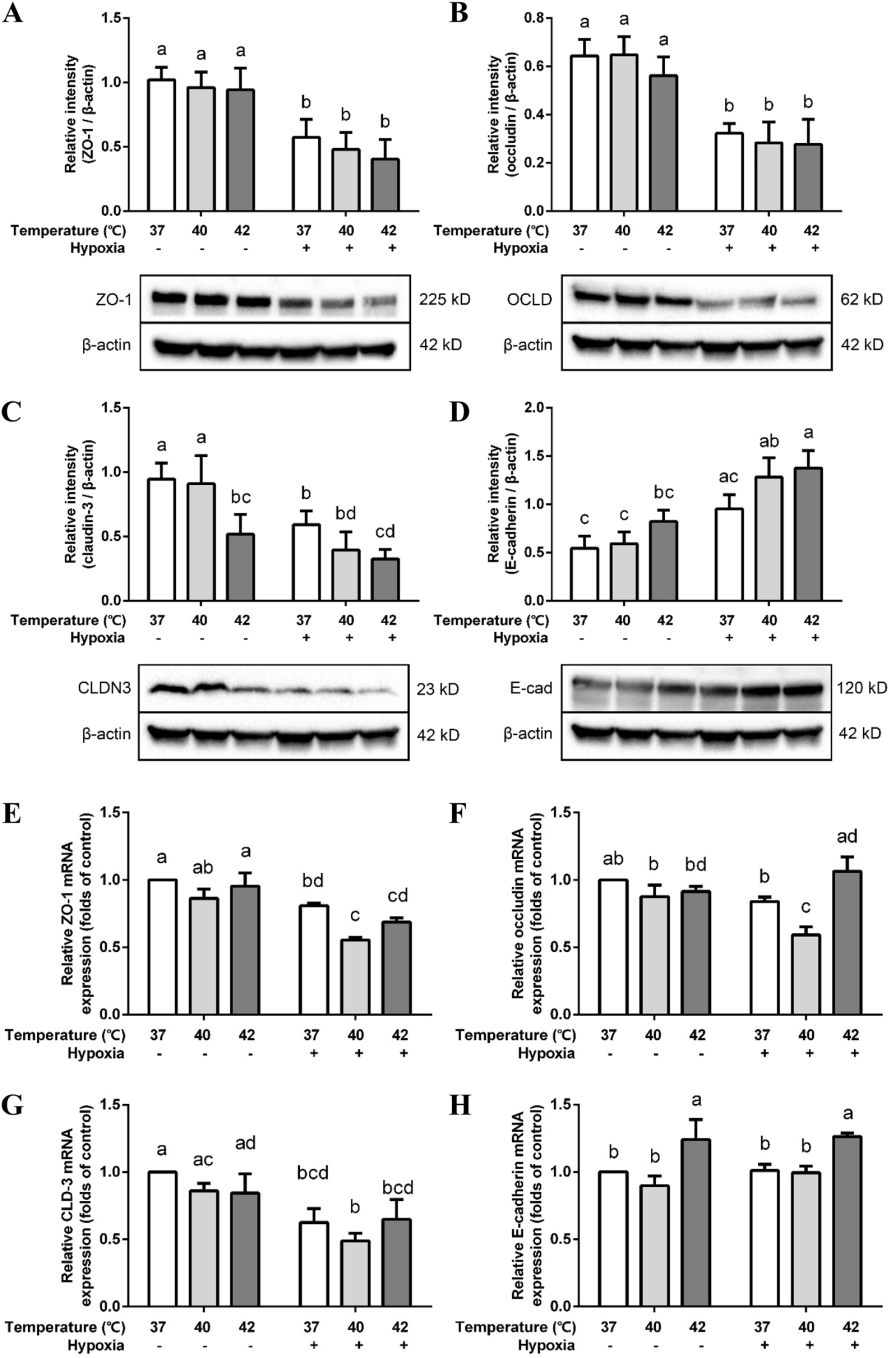
Effects of hypoxia and/or heat treatment on TJ/AJ proteins in the co-culture model. Relative protein expression of ZO-1 (**A**), occludin (**B**), claudin-3 (**C**) and E-cadherin (**D**) assessed by Western Blot. All target proteins were normalized to reference protein β-actin. ZO-1 (**E**), occludin (**F**), claudin-3 (**G**) and E-cadherin (**H**) mRNA levels were assessed by qRT-PCR. The target genes were normalized to housekeeping gene β-actin. All values were presented as means ± SD (N = 3, n = 3). Statistical differences were analyzed by two-way ANOVA followed by the Bonferroni’s multiple comparison test. Means without a common letter differ at *p* < 0.05. *OCLD* occludin; *CLDN3* claudin-3; *E-cad* E-cadherin.

Considering the significant drop of TEER values and increased Lucifer Yellow flux after 2 h of hypoxia/heat exposure, the status of the cell–cell junctional complexes was investigated. Three key TJs proteins—ZO-1, occludin (OCLN) and claudin-3 (CLDN3)– and the most essential epithelial AJ protein, E-cadherin, were analyzed. The protein levels of all the tight junctions were decreased by hypoxia (Fig. 4A–C), but remained unaffected by heat treatment, except for claudin-3. Claudin-3 was significantly down-regulated at 42 ℃ under normoxia and hypoxia conditions (Fig. 4C). In contrast, E-cadherin protein expression was significantly upregulated when heat treatment was combined with hypoxia (Fig. 4D).

ZO-1 and claudin-3 mRNA transcription levels were down-regulated by hypoxia with and without heat treatment (Fig. 4E,G), while occludin mRNA was only significantly down-regulated when hypoxia treatment was combined with 40 ℃ (Fig. 4F).

E-cadherin mRNA expression was enhanced at 42 ℃ under hypoxia as well as normoxia conditions, but no additional effect of hypoxia was observed (Fig. 4H). However, all the changes in mRNA expression were less than two-fold.

### Heat treatment and/or hypoxia changes TJs distribution in the co-culture model

**Figure 5:**
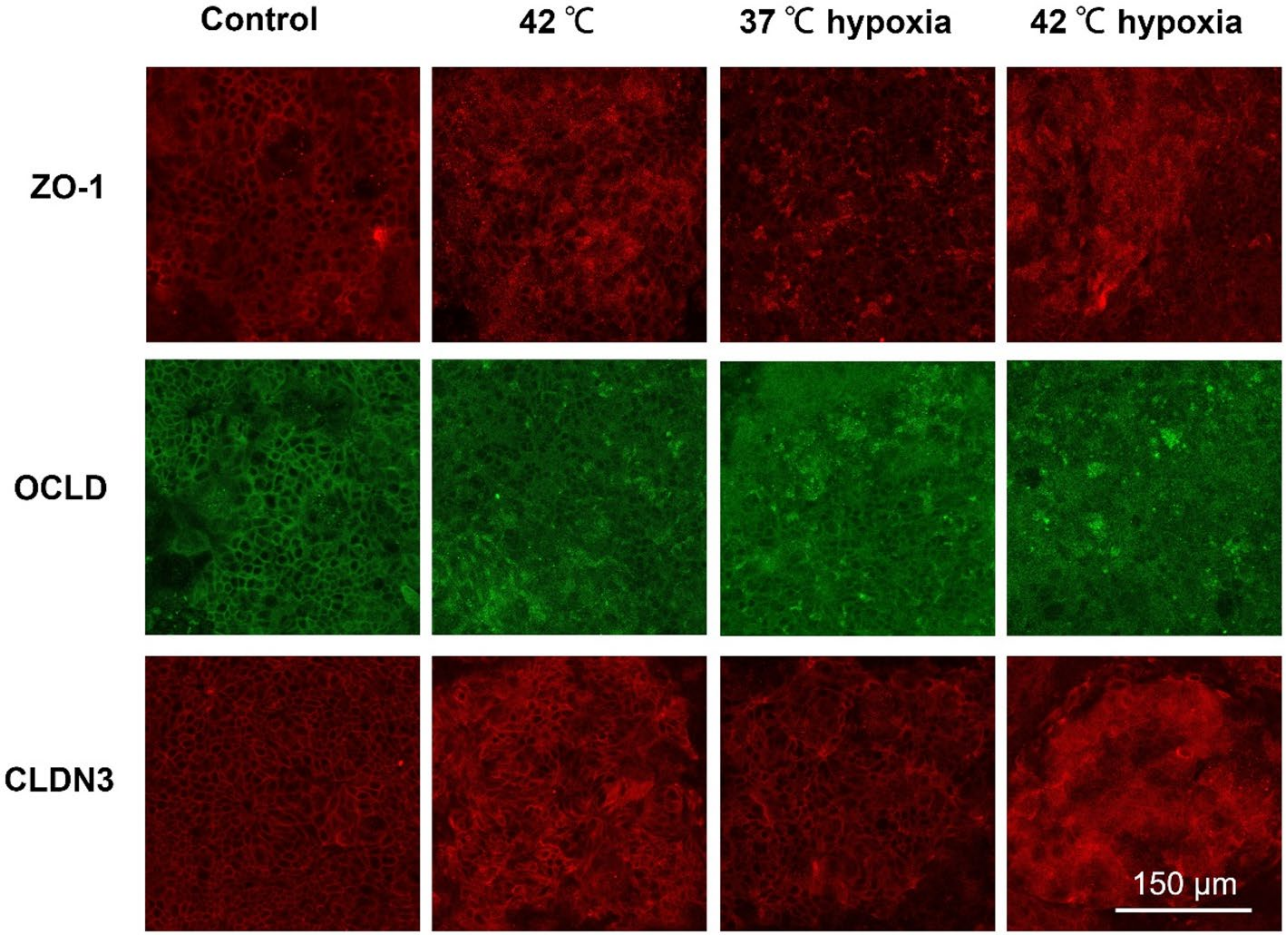
Effects of hypoxia and/or heat treatment on TJ localization in the co-culture model. Red or green color: anti-ZO-1, anti-OCLD or anti-CLDN3 antibody conjugated with Alexa-Fluor fluorescent secondary antibodies. The images were acquired by Leica TCS SP8 microscope with HCX IRAPO L 25 × /0.95 objective lens at 1.6 × digital magnification, pinhole: 1.5 AU. *OCLD* occludin; *CLDN3* claudin-3.

Immunofluorescent staining for all TJs, CLDN-3, OCLN and ZO-1, were conducted to clarify their localization in the epithelial cell layer. In the intact Caco-2/HT-29 cell layers, the TJ are localized at the cell membrane and showed continuous belt-like structures. HS-exposed cells exhibited disturbed cell junctions (CLDN-3, OCLN and ZO-1) and the belt-like structures are less clear to observe, especially at 42 ℃, which might indicate the translocation of these TJ proteins (Fig. 5).

Two hours of hypoxia exposure also induced a disturbed and irregular cellular distribution of the cell junctions in Caco-2/HT-29 co-cultures as observed in concentrated TJ clusters and a more diffuse and cytosolic pattern. This pattern seemed to increase when hypoxia is combined with higher temperatures (Fig. 5). Isotype control and secondary antibody control of the immunofluorescence staining are depicted in Figure S4.

### Heat treatment increases mucin-related gene transcription in the co-culture model

**Figure 6:**
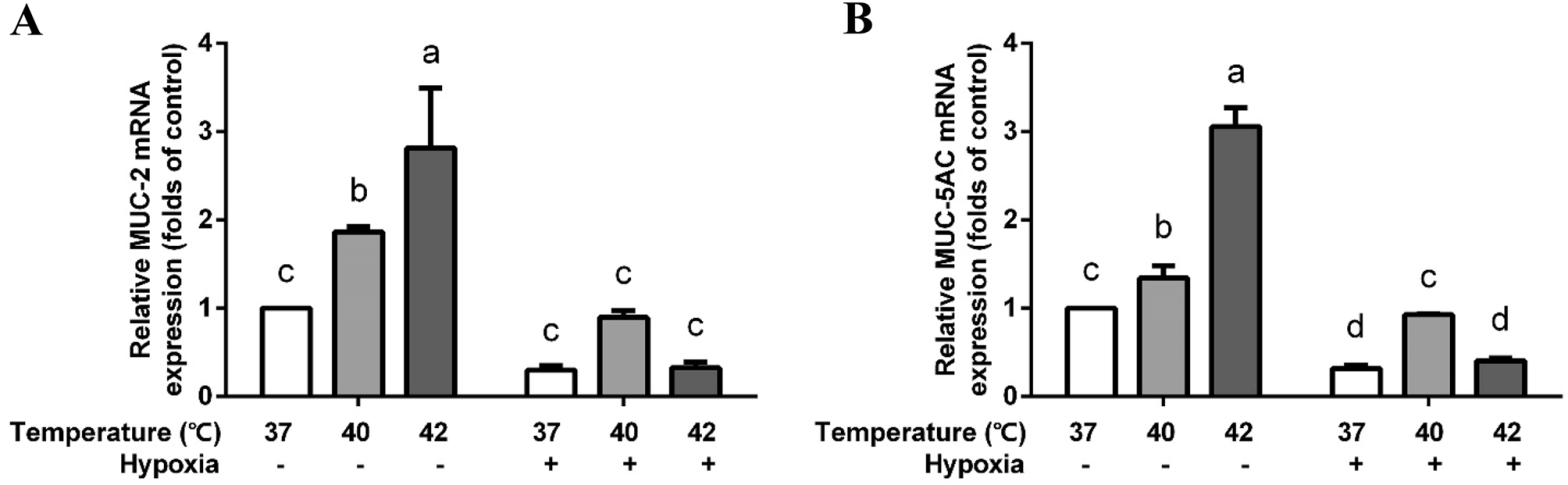
Effects of hypoxia and/or heat treatment on mucin gene production in the co-culture model. MUC-2 (**A**) and MUC-5AC (**B**) mRNA levels were assessed by qRT-PCR and normalized to the housekeeping gene β-actin. All values were presented as means ± SD (N = 3, n = 3). Statistical differences were analyzed by two-way ANOVA followed by the Bonferroni’s multiple comparison test. Means without a common letter differ at *p* < 0.05.

Mucin-related genes, including MUC-2 and MUC-5AC, were detected after the hypoxia and heat exposure. Under normoxia, both MUC-2 and MUC-5AC genes transcription were enhanced by high temperatures. Interestingly, this enhancement was attenuated by hypoxia (Fig. 6).

## Discussion

In this study, we established an in vitro co-culture model of Caco-2 and HT-29 cells and investigated the effect of hypoxia and/or HS challenges on intestinal epithelium by measuring intestinal barrier function, the heat shock response and oxidative stress response.

Caco-2 and HT-29 co-culturing offers a simple and standardized methodology to reach morphofunctional features similar to the in vivo human intestinal epithelium. Because of the physiological similarity and effectivity, this co-culture model has been used to investigate chemical resorption and bacteria adhesion and invasion^26, 29^. The percentage of these two cell types, absorptive cells and mucus secreting cells, fluctuates along the different gut regions. In general, Goblet cells make up 10–24% of total enterocytes^30^. Ferraretto et al*.* investigated the co-culture model and demonstrated that 7:3 (Caco-2:HT-29) is the most optimal ratio for seeding the cells compared to ratio 1:1 and 3:7^31^, as observed by changes in microvilli, junctional apparatus and mucus granules. Pan et al*.* proved that 9:1 (Caco-2:HT-29) is the optimal ratio in the in vitro model for permeability studies^32^, which is in agreement with our study, since in the 9:1 (Caco-2:HT-29) ratio model, mucins were produced and TEER values corresponded to the in vivo human colonic epithelium (300–400 Ω × cm^2^)^33^. Moreover, after 2-h of hypoxia and heat stress, TEER values and Lucifer Yellow permeability did not change in the separate cell lines while these parameters did change significantly in the co-culture model, which suggest that both cell lines are crucial for the observed effects in this in vitro model.

Heat shock proteins are a highly genetically conserved family of molecules, which are critical for the stress resilience in organisms. While their synthesis process was initially described in relation to elevated temperatures, investigations showed that the generation of heat shock proteins can be induced by multiple stress stimuli including UV light, drought, radioactivity and even mechanical injury. Hypoxia is also reported as an acute or chronic inducer of HSP^34, 35^. In this study, it was demonstrated that 2 h of hyperthermia exposure increased expression of HSP-70 and its transcription factor HSF-1. While in combination with hypoxia this was further increased. Among the hypotheses for cross-tolerance between heat and hypoxic acclimation, the best supported one is the interaction between HSPs and HIF, although the exact mechanism is not fully elucidated. The increased HSP response to heat resilience can independently trigger the HIF-1α pathway and vice versa^36^. Hyperthermia and sublethal hypoxia pre-treatment increase HSP-70 expression in rat retinal ganglion cells and enhanced tolerance to anoxia and excitotoxicity^37^. Similarly, increased HIF-1α expression by hyperthermia acts as a danger signal, which allows the organs to anticipate a probable worse situation—subsequent occurrence of hypoxia. The increased HIF-1α expression in heart tissue following heat acclimation lead to enhanced tissue tolerance to ischemia^36^. Especially, (long term) strenuous exercise or work at high altitude will lead to the cross acclimation between hyperthermia and hypoxia, which may confer cross-adaptive advantages in response to continuous heat shock or hypoxic conditions.

However, in this study, we did not observe a significant difference in either HIF-1α expression or Nrf2 nuclear translocation between hyperthermia or normal temperature-exposed cells. This may be explained by the short duration of the heat incubation. Similar results were found by Assayag et al*.*, who demonstrated that chronic (30 d), but not acute heat acclimation (2 d), contributed to a cytoprotective effect related to ischemic-reperfusion stress^38^. Moreover, the presence of hypoxia only elevated HSP-70 mRNA transcription, while no significant changes were observed in protein expression. Since 2 h are the maximal non-toxic hypoxic exposure time in the co-culture model used in this study, a proper long-term animal study is necessary to elucidate the interaction between the oxidative stress response (e.g. HIF) and heat shock response (e.g. HSPs).

Besides interaction with HIF, HSP-70 itself is an important modulator of barrier function. Our previous study suggested that α-lipoic acid stimulates intestinal epithelial recovery from HS by enhancing HSP-70 expression^20^. Similarly, studies proved that either HSP-70 protein enhancement or exogenous HSP-70 significantly improved intestinal barrier integrity^39, 40^. This effect can be mediated by HSF-1 phosphorylation or direct interaction with ZO-1^41^. Interestingly, HSPs are regarded as extracellular signaling biomarkers and immunomodulant by more and more researchers, since they are secreted to the cell exterior and take part in immunomodulation^42–44^. This indicates that in some inflammatory diseases that are accompanied by long-term low fever (such as tuberculosis or virus infection) or local hypoxia (such as COPD and asthma), HSPs may be a novel modulatory target. Moreover, the Nrf2-Keap1 pathway is also responsible for maintaining intestinal epithelial integrity under ongoing oxidative stress status^45^. In this study we demonstrated that after hypoxia treatment Nrf2 was not only translocated from cytoplasm into the nucleus, but also actively synthesized in the plasma to restore homeostatic levels. It can be suggested that beyond serving as a transcription factor, Nrf2 may also act as a modulator to another receptor protein.

The intestinal mucosal barrier is the frontier defending human body against multiple stimuli. It consists of a mechanical barrier, biochemical barrier and immune barrier^46, 47^. TJs and AJs assembling in the cytomembrane delicately ensure the tightness of the mechanical barrier. This study demonstrated that hypoxia decreased the expression and distorted the distribution of ZO-1, claudin-3 and occludin in the co-culture model. However, the protein and mRNA expression of these TJs is not significantly changed after 2 h of 40 ℃ exposure. Our previous study showed that, even after 24 h of hyperthermia, the protein expression of these TJs did not change in a Caco-2 monolayer^48^. This could be related to the chaperone function of HSPs. Caco-2 cells cultured for 2, 4 and 8 h in a hypoxic environment significantly decreased the expression of occludin and ZO-1^49^, which implies that under pathological conditions, hypoxia is an important mechanism leading to dysfunction of the intestinal epithelial barrier. Notably, E-cadherin was elevated when temperature increased and hypoxia was introduced, which indicates a potential compensatory mechanism behind the disruption of barrier integrity when interacting with stress stimuli. Similarly, HO-1 mediated the increase of claudin-4 to compensate the loss of TJ integrity in Caco-2 cells after hypoxia treatment^49^.

Besides the mechanical barrier, the biochemical barrier formed by a mucus layer, is also an important frontier which protects the intestine against environmental stressors. MUC-2 and MUC-5AC are two types of human mucins, forming highly glycosylated regions that are hydrophile and able to maintain the high water content^50^. Mucous layer serves as a buffer layer and is crucial to protect the epithelium, as MUC-2 knock-out mice spontaneously develop colitis^51^. However, Caco-2 cells are reported as non/low-mucus producing cells and do not or hardly express mucin-related genes, the co-culture model with HT-29 allows us to investigate the contribution of mucin-producing cells as well^25^. We showed that both MUC-2 and MUC-5AC gene transcription was enhanced by high temperatures. Heat stress induces mucus hypersecretion in mice airways, which may be related to the up-regulation of EGFR and down-regulation of AQP5^52^. HSP-70 is also possibly responsible for the cytoprotective effects on the mucus layer of the stomach^53^. Hypoxia up-regulates MUC-5AC by the HIF-1α signalling pathway in human nasal epithelia and human bronchial epithelia^54, 55^, while we observed that the MUC-2 and MUC-5AC mRNA levels are attenuated by hypoxia in the intestinal epithelial co-culture model . More research is needed to unravel the mechanism behind MUC-2 and MUC-5AC mRNA changes in intestinal epithelial cells induced by heat stress and hypoxia.

The disturbed integrity of the intestinal barrier by hyperthermia and hypoxia may have clinical consequences, since a leaky gut has been demonstrated in several (chronic) stress conditions. For example, strenuous exercise, such as cycling and marathon leads to an continuous increased body temperature and redistribution of circulating blood from core organs to skeletal muscles, resulting in local hypoxia and heat stress. It has been reported that endurance exercise caused impairment of intestinal integrity in humans^56, 57^.

In conclusion, after successful establishment of the Caco-2/HT-29 co-culture system, resembling the human situation, we investigated the effect of hyperthermia and/or hypoxia exposure on intestinal barrier function, heat shock response and oxidative stress response. HSP-70 and HSF-1 protein levels were up-regulated by hyperthermia and/or hypoxia, while HIF-1α and Nrf2 protein levels were only increased after hypoxia exposure. Related to intestinal barrier function, epithelial permeability was increased when hyperthermia was combined with hypoxia. ZO-1, claudin-3 and occludin expression levels were decreased by hypoxia, while E-cadherin protein expression was enhanced by hypoxia. The distribution of TJ protein was disturbed by hyperthermia and/or hypoxia. MUC-2 and MUC-5AC genes transcription levels were only up-regulated by hyperthermia. This Caco-2/HT-29 co-culture model can be used to investigate possible interventions to reverse hyperthermia and/or hypoxia-induced intestinal epithelial injury.

## Material and methods

### Cell culture

Caco-2 (ATCC HTB-37, USA) and HT-29 (ATCC HTB-38, USA) cells were separately seeded at a density of 3.5 × 10^5^ cells per 75-cm^2^ flask. The cells were grown in tissue culture flasks at 37 °C, 5% CO_2_ and 90% relative humidity environment. The cells were sub-cultured at ~ 90% confluence (∼6 days) by 0.25% trypsin and 0.02% EDTA solution. The culture medium (DMEM supplemented with 10% fetal calf serum, 1% non-essential amino acids, 1% L-glutamine and 1% penicillin and streptomycin) was refreshed every other day.

### Cell co-culture

Caco-2 and HT-29 cells were counted by an automated cell counter (Cellometer Auto T4, Nexcelom Bioscience, USA), mixed evenly in different proportions (Caco-2 : HT-29 = 1:0, 9:1, 3:1, 1:1, 1:3, 1:9 and 0:1) and seeded into the apical chambers of 24-well Transwell inserts (Corning, USA) with a final density of 1 × 10^5^ cells/cm^2^ in each insert. Cells were cultured under the same atmosphere as described in 2.1. and allowed to grow for 17 days. The medium (300 μl in the apical chamber and 700 μl in the basolateral chamber) was refreshed every other day.

### Hypoxia and/or heat stress inducement

Caco-2 and HT-29 co-cultures with TEER values > 300 Ω × cm^2^ which reached a plateau (~ 17 days after seeding) were used for further experiments. Hypoxia was induced by using a multi-functional incubator (Galaxy 48R, Eppendorf AG, Germany) at different incubation durations (15 min to 24 h). The O_2_ concentration in the chamber was maintained at 5%^58, 59^, with a residual gas mixture composed of 5% CO_2_ and balanced N_2_. For heat stress inducement, the environmental temperature was set at 37 ℃ (control), 40 ℃ or 42 ℃. The relative humidity was kept at 90%. During the hypoxia and/or heat stress inducement, volume of the medium was remained 100 μl to facilitate the gas exchange.

### Trans-epithelial electrical resistance (TEER) value

Referring to the method of Yamashita et al*.*^60^, the cell monolayer integrity was determined by TEER measurement using an epithelial volt-ohm meter (Millicell ERS-2, Merck, USA). The chopstick electrodes were placed into two chambers of each Transwell insert. TEER values > 300 Ω × cm^2^ were regarded as valid for further permeability studies.

### Lucifer yellow permeability test

The fluorescent chemical Lucifer Yellow (LY, 0.44 kDa, Sigma-Aldrich, USA) was used to measure the paracellular permeability across the co-culture monolayer. At the end of hypoxia/HS challenge, 30 μl LY (200 μg/ml) was added into the apical chambers of Transwell inserts. The inserts were kept at 37 °C in dark for 4 h, then the medium from the basolateral chamber was collected for fluorescent intensity assay (λ_ex_ 428 nm, λ_em_ 540 nm) using a fluorometer (Fluoroskan Ascent FL, Thermo Fisher Scientific, USA). The fluorescent emission intensity was converted into fluorescein flux per hour by standard curve.

### Alcian blue/nuclear fast red staining

Acid mucin and mucopolysaccharides produced by HT-29 cells were determined by Alcian Blue staining. Co-cultured cells fixed with 10% formalin solution were acidized with 3% acetic acid for 3 min then stained with 1% Alcian Blue (Sigma-Aldrich, USA) solution (in acetic acid, pH 2.5) for 30 min at room temperature. After being gently rinsed with 3% acetic acid well, the cells were rinsed with distilled water then counterstained with 0.1% Nuclear Fast Red (Sigma-Aldrich, USA) solution (in 5% aluminum sulfate solution) for 5 min. After counterstaining, the cells were rinsed well with distilled water, dehydrated with 95% ethanol followed by 100% ethanol, cleared with xylene, and mounted with Calbiochem mounting solution (#345789, Millipore, USA). The cover-slipped cells were imaged by inverted microscope system (Eclipse Ts2-FL, Nikon, Japan).

### Cytotoxicity assay

After specific exposure to hypoxia and/or high temperature, the supernatant was collected and immediately assayed for cytotoxicity using CytoTox 96 Cytotoxicity Non-Radioactive Assay kit (Promega, USA). The results were presented as lactate dehydrogenase (LDH) production, indicating cytomembrane damage and intracellular contents release. The group in which all cells were intentionally lysed by lysis buffer was included as a positive control.

### RNA isolation and quantitative real-time PCR (qRT-PCR)

Cells were harvested and lysed by using SV Total RNA Isolation System (Promega, USA) according to the manufacturer’s protocol. The concentration and integrity of isolated RNA was quantified by using NanoDrop 1000 spectrophotometer (Thermo Fisher Scientific, USA) and checked by 1.5% agarose gels electrophoresis then reverse-transcribed into cDNA using iScript cDNA Synthesis kit (Bio-Rad, USA) and T100 Thermal Cycler (Bio-Rad, USA). The 18S and 28S ribosomal RNA bands indicating the intact RNA samples were clearly visible in both the control and model groups (Figure S5), which indicates that 2 h of hypoxia and/or heat exposure did not degrade total RNA integrity.

For qRT-PCR, 15 μl mixture containing 6 μl diluted cDNA templates, 7.5 μl iQ SYBR Green Supermix (Bio-Rad, USA), 0.6 μl forward primer (100 μM), 0.6 μl reverse primer (100 μM) and 0.3 μl nuclease-free water was prepared for each reaction. The amplification was performed using C1000 Thermal Cycler (Bio-Rad) and analyzed by CFX Manager system (version 3.1, Bio-Rad, USA).

The specific primers listed in Table 1 were commercially manufactured (Biolegio BV, Nijmegen, the Netherlands) and used after confirmation of primer annealing and subsequent melting curve analysis. Cycling conditions for qRT-PCR amplification were 95 °C for 3 min and then 35 cycles of denaturation at 95 °C for 10 s, annealing for 30 s and extension at 72 °C for 30 s. The housekeeping gene (β-actin) from each sample was loaded in the same plate as its target gene and assessed simultaneously with each reaction and used for further standardization. The results were compared by the Ct value of the target genes using the formula 2^ΔΔCT^. The mean of control groups was regarded as 1 and all other groups were expressed as “folds of control”.

**Table 1:** Sequences of the used primers. AT: annealing temperature.

Gene or coded protein	Primer sequence (5′ → 3′)		AT (℃)	Reference
	Forward	Reverse		
β-actin	TTGTTACAGGAAGTCCCTTGCC	ATGCTATCACCTCCCCTGTGTG	56	NM_001101.3
claudin-3	CTGCTCTGCTGCTCGTGTC	CGTAGTCCTTGCGGTCGTAG	63	NM_001306
E-cadherin	TGGACCGAGAGAGTTTCCCT	CCCTTGTACGTGGTGGGATT	60	BC144283.1
HIF-1α	CCAGCAGACTCAAATACAAGAACC	TGTATGTGGGTAGGAGATGGAGAT	61	NM_001530
HO-1	CCAGCGGGCCAGCAACAAAGTGC	AAGCCTTCAGTGCCCACGGTAAGG	62.6	X06985.1
HSP-70	AGAGCCGAGCCGACAGAG	CACCTTGCCGTGTTGGAA	57	NG_011855.1
MUC2	CAGCACCGATTGCTGAGTTG	GCTGGTCATCTCAATGGCAG	59	^61^
MUC5AC	GCATCCAGCTCTGTGGCTTA	CACTGTCAACCCCTCTGACC	59	^61^
occludin	TTGGATAAAGAATTGGATGACT	ACTGCTTGCAATGATTCTTCT	57	NM_002538
ZO-1	GAATGATGGTTGGTATGGTGCG	TCAGAAGTGTGTCTACTGTCCG	55.8	NT_010194.17

### Total, nuclear and cytoplasmic protein extraction

For total protein extraction, co-cultured cells exposed to HS/hypoxia for 2 h were lysed immediately after removal from the incubator using 50 μl Pierce RIPA buffer (Thermo Fisher Scientific, USA) containing protease inhibitor cocktail (#11836170001, Roche, Switzerland). Total protein concentration was assessed and standardized with Pierce BCA Protein Assay Kit (Thermo Fisher Scientific, USA). The protein samples used for determining HIF-1α expression were preincubated with dimethyloxalylglycine (DMOG) (Sigma-Aldrich, USA), a HIF prolyl-hydroxylase inhibitor, which stabilizes HIF-1α during the normoxic lysis procedure.

Nuclear and cytoplasmic proteins were extracted using NE-PER Nuclear and Cytoplasmic Extraction Reagents (Thermo Fisher Scientific, USA) and standardized by using Pierce BCA Protein Assay Kit (Thermo Fisher Scientific, USA).

### Western blot analysis

Equal amounts (20 μg) of boiled protein samples were separated by electrophoresis (Criterion Gel, 4–20% Tris–HCL, Bio-Rad, USA) and electrotransferred onto Trans-Blot Turbo polyvinylidene difluoride (PVDF) membranes (midi format 0.2 μm, Bio-Rad). After being blocked with 5% skimmed milk (in PBS containing 0.05% Tween-20 (PBST)), the membranes were incubated overnight at 4 ℃ with the primary antibodies of HSP-70 (1:1000, #C92F3A-5, Enzo life science, Belgium), HSF-1 (1:1000, #D3L8I, Cell Signaling, USA), HIF-1α (1:2000, #ab113642, Abcam, UK), Nrf2 (1:1000, #D1C9, Cell Signaling, USA), ZO-1 (1:2000, #ab190085, Abcam, UK), occludin (1:500, #71–1500, Invitrogen, USA), claudin-3 (1:1000, #341700, Invitrogen, USA) and E-cadherin (1:1000, #610182, BD sciences, USA), then incubated with correspondent horseradish peroxidase (HRP) -conjugated secondary antibodies (1:10,000, Dako, Denmark) for 2 h at room temperature. After being rinsed well with PBST and incubated with ECL detection reagent (#RPN2235, GE Healthcare, USA), the membranes were exposed to ECL imaging system (ChemiDoc MP, Bio-Rad, USA). The optical intensity of the blots was recorded and analyzed by using Image Lab (version 6.01, Bio-Rad, USA) and ImageJ (version 1.80, NIH, USA) software. Housekeeping proteins β-actin (1:2000, #13E5, Cell Signaling, USA) and histone H3 (1:1000, #ab18521, Abcam, UK) were used as reference to total proteins and nuclear proteins. The blots were cut prior to hybridization with the antibodies to simultaneously detect various target proteins with different protein sizes on the same membrane. The blots on the same membrane were stripped by Restore PLUS Western Blot Stripping Buffer (Thermo Fisher Scientific, USA) and re-blotted.

### Immunofluorescent staining

Subcellular localization of TJ/AJ proteins was determined by immunostaining. Co-cultured cells in the inserts fixed with 10% formalin solution were permeabilized with 0.1% Triton-X-100 (in PBS) for 5 min, followed by blocking with 5% goat serum (in PBS containing 1% bovine serum albumin (BSA)) for 30 min at room temperature. Thereafter, the cells were incubated for 2 h at room temperature with the primary antibodies of ZO-1 (1:100, #ab190085, Abcam, UK), occludin (1:50, #71–1500, Invitrogen, USA) and claudin-3 (1:100, #341700, Invitrogen, USA) followed by incubation with Alexa-Fluor fluorescently conjugated secondary antibodies (1:1000, Invitrogen, USA) for 1 h at room temperature. The nuclei were counterstained with Hoechst 33,258 (1:2000, Invitrogen, USA) or ProLong Gold Antifade Mountant with DAPI (#P36935, Invitrogen, USA). After Hoechst counterstain, the cells were rinsed well and mounted with Calbiochem anti-fade mounting solution (#345789, Millipore, USA). The cover-slipped cells were visualized by confocal microscopy system (TCS SP8, Leica, Germany). Rabbit IgG polyclonal isotype control (#ab176094, Abcam, UK) was used for occludin and claudin-3 antibodies and goat IgG polyclonal isotype control (#AB108C, R&D Systems, USA) was used for ZO-1 antibody.

### Statistical analysis

Results are expressed as means ± SEM of 3 independent experiments (N = 3), each performed in triplicate (n = 3) unless otherwise stated. Statistical analyses were performed by using GraphPad Prism (version 6.04, GraphPad). Differences between groups were determined by using Two-way analysis of variance (ANOVA), with Bonferroni post-hoc test. Results were considered statistically significant when *p* < 0.05.

## Supplementary Information

Supplementary Information.
