# Supplementary material for: Hypoxia and heat stress affect epithelial integrity in a Caco-2/HT-29 co-culture
Source: Sci Rep. 2021 Jun 23;11:13186. doi: 10.1038/s41598-021-92574-5 (PMC8222227; doi:10.1038/s41598-021-92574-5)

Supplementary Information to

**Hypoxia and heat stress affect epithelial integrity in a**

**Caco-2/HT-29 co-culture**

**Puqiao Lian^1^, Saskia Braber^1^, Soheil Varasteh^1^, Harry J. Wichers^2^, Gert Folkerts^1, *^**

^1^ Division of Pharmacology, Utrecht Institute for Pharmaceutical Sciences, Faculty of Science, Utrecht University, Utrecht, The Netherlands

^2^ Food & Biobased Research, Wageningen University & Research, Wageningen, The Netherlands

* **Corresponding author:** G.F. ([g.folkerts@uu.nl](mailto:g.folkerts@uu.nl))

Utrecht University, Department of Pharmaceutical Sciences, Division of Pharmacology

Universiteitsweg 99, 3584 CG, Utrecht, The Netherlands


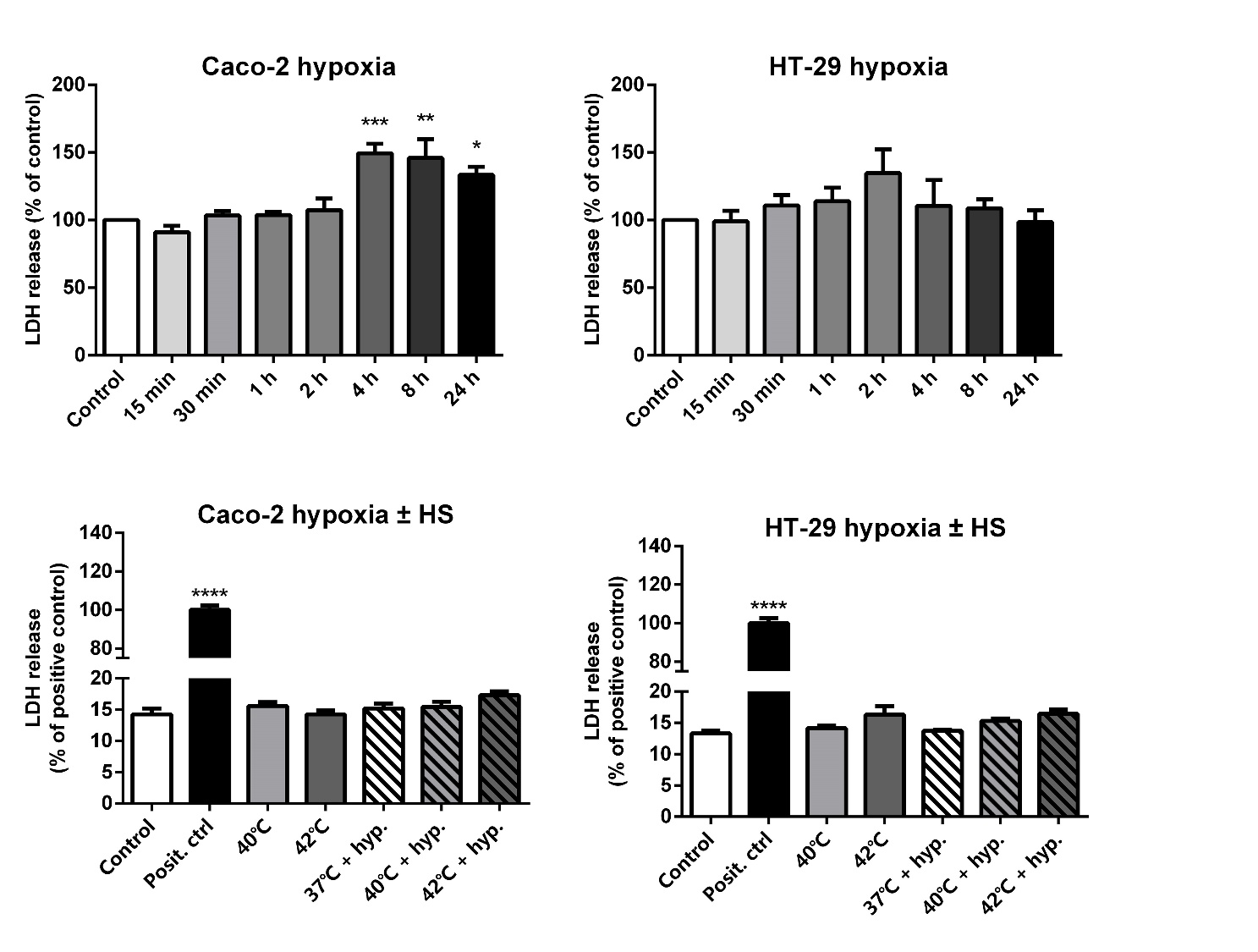


**A**

**B**

**C**

**D**

**Figure S1.** **(A)** LDH release of Caco-2 cells after different periods of hypoxia exposure. **(B)** LDH release of HT-29 cells after different periods of hypoxia exposure. **(C)** LDH release of Caco-2 cells after 2 hours of hypoxia ± heat treatment. In the positive control group, the cells were lysed with lysis buffer. **(D)** LDH release of HT-29 cells after 2 hours of hypoxia ± heat treatment. All values were presented as means ± SEM (N=3, n=3). Statistical differences were analyzed by Two-way analysis of variance (ANOVA), with Bonferroni post-hoc test. *p<0.05, **p<0.01, ***p<0.001, **** p<0.0001 vs control. Posit. ctrl: positive control; hyp.: hypoxia.


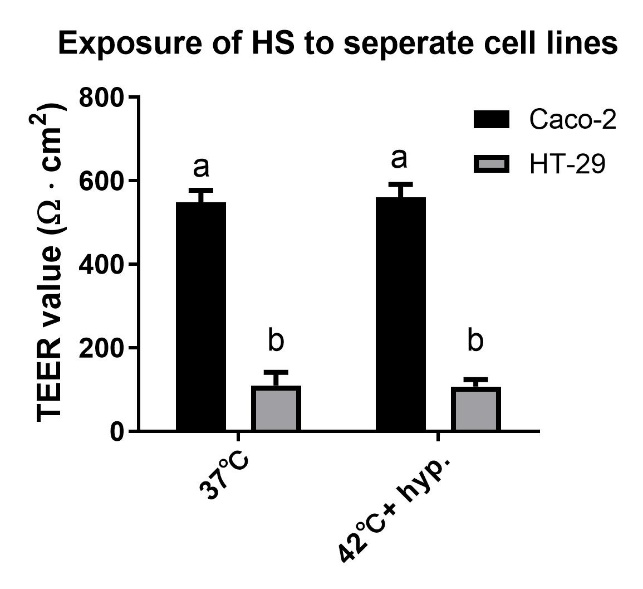


**A**


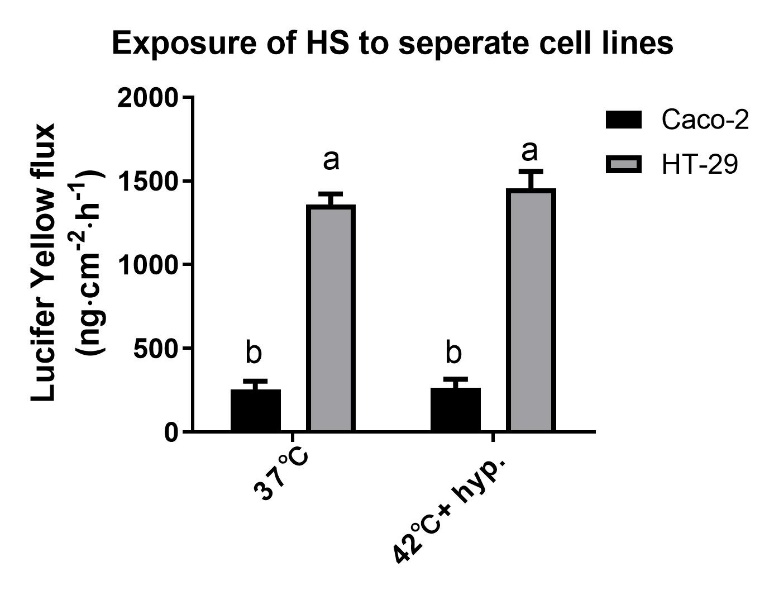


**B**

**Figure S2.** The effect of 2 hours of hypoxia and hyperthermia on the intestinal integrity of Caco-2 or HT-29 cell lines. **(A)** TEER values of Caco-2 or HT-29 cells before and after 2 hours of hypoxia ± heat treatment. **(B)** Lucifer Yellow permeability of Caco-2 or HT-29 cells before and after 2 hours of hypoxia ± heat treatment. All values were presented as means ± SEM (N=3, n=3). Statistical differences were analyzed by two-way ANOVA followed by the Bonferroni’s multiple comparison test. Means without a common letter differ at p<0.05. Hyp.: hypoxia.


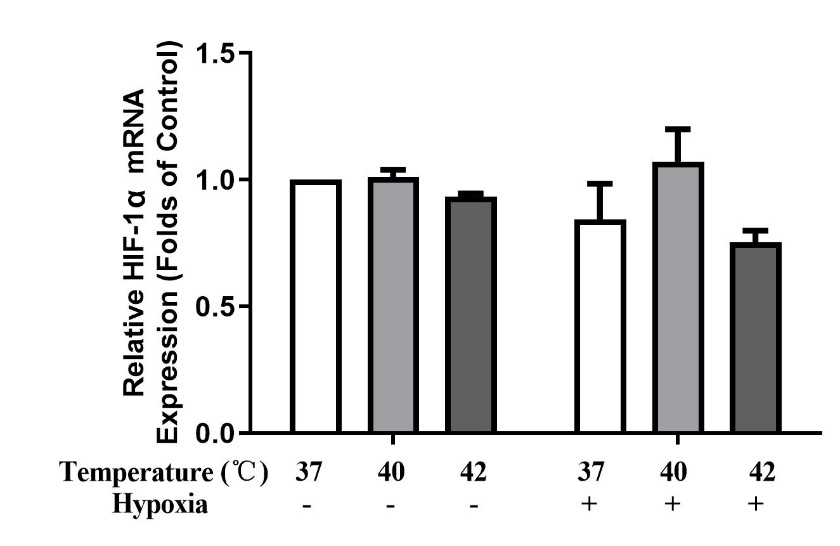


**Figure S3.** HIF-1α mRNA levels assessed by qRT-PCR. The target genes were normalized with housekeeping gene β-actin. All values were presented as means ± SD (N=3, n=3). Statistical differences were analyzed by two-way ANOVA followed by the Bonferroni’s multiple comparison test.


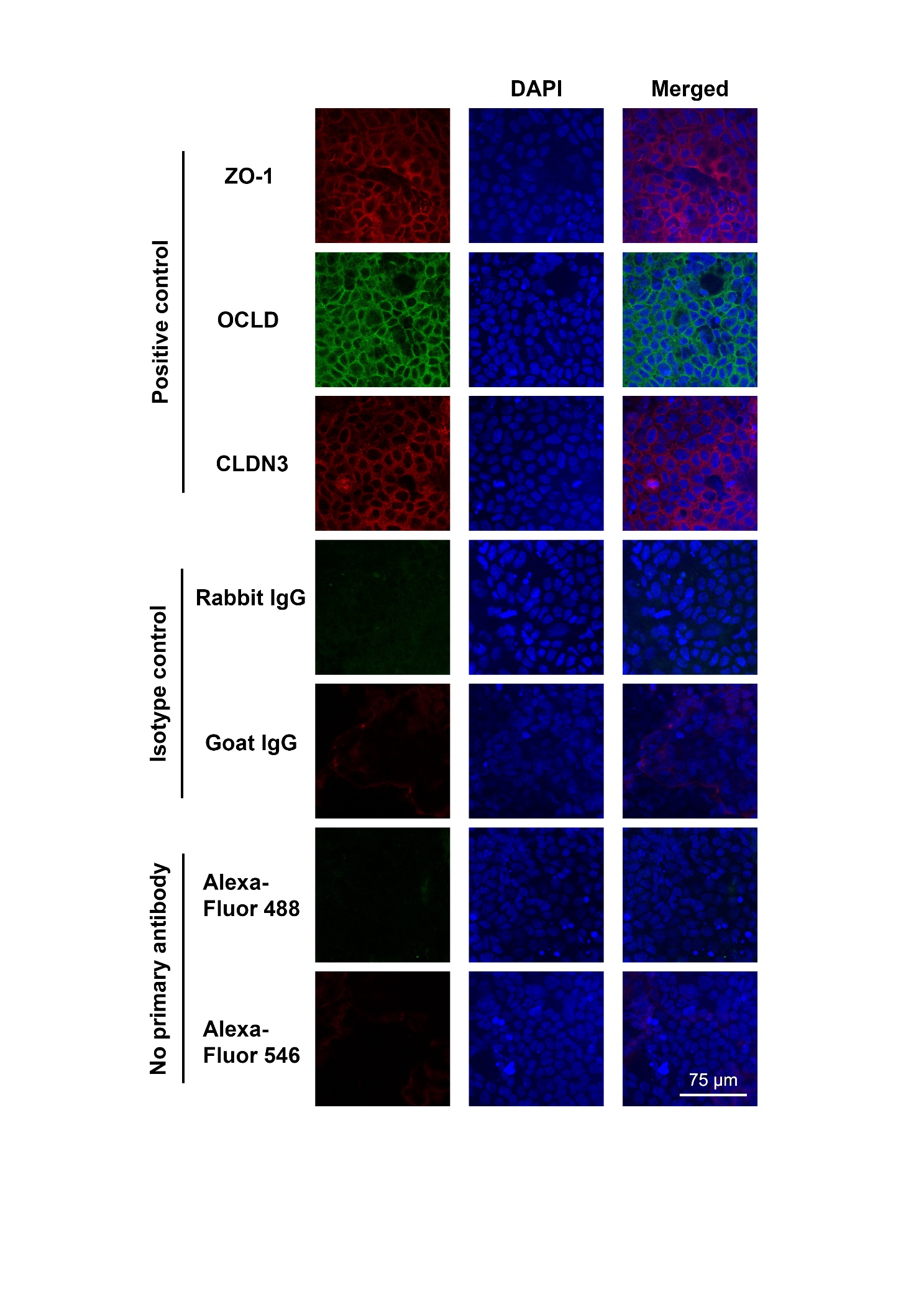


**Figure S4**. Isotype control and secondary antibody control of the immunofluorescence staining. Red or green color: signals from Alexa-Fluor^®^ 488 or Alexa-Fluor^®^ 546 fluorescent secondary antibodies; blue color: DAPI located nuclei. The results were acquired by Leica TCS SP8 microscope with HCX IRAPO L 25×/0.95 objective lens at 3.2× digital magnification, pinhole: 1.5 AU. OCLD: occludin; CLDN3: claudin-3.


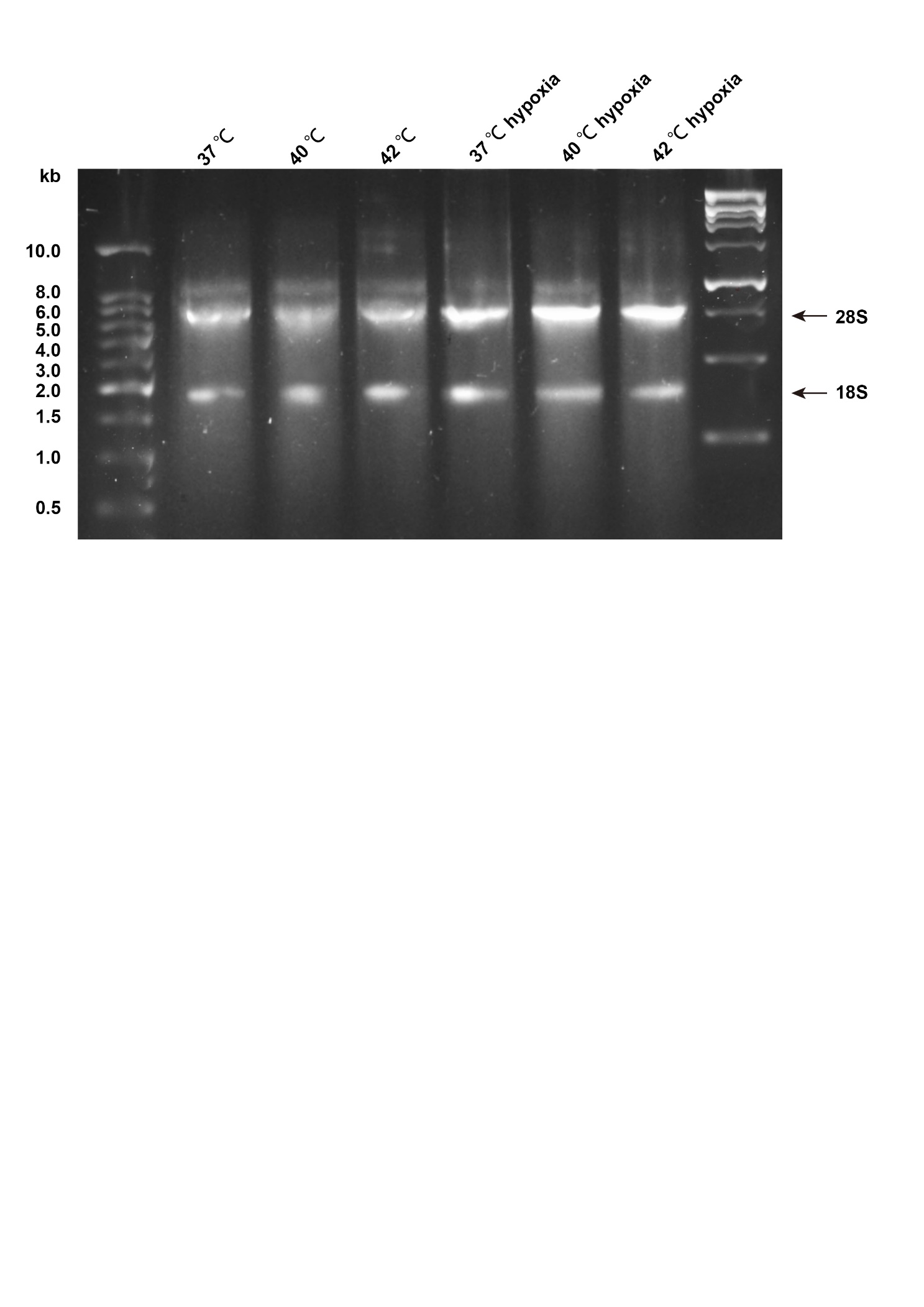


**Figure S5**. The RNA integrity analysis by 1.5% agarose gel electrophoresis and the indicated 28S/18S RNA bands.


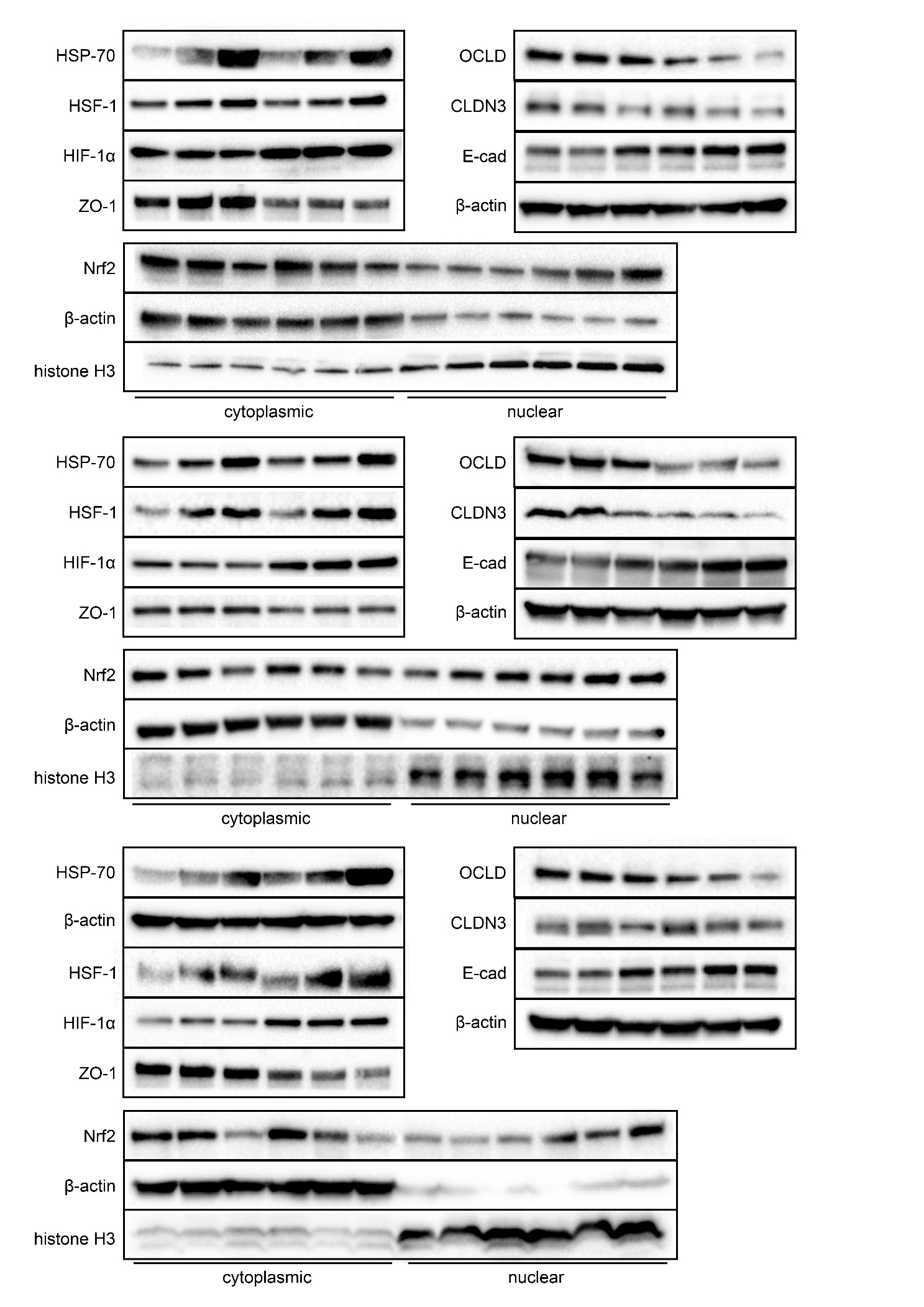


**A**

**B**

**C**

**Figure S6:** Western blots images used for quantification of the three independent repeats **(A, B** and **C)**. The blots on the same membrane were stripped, re-blotted and referred to the same reference protein (β-actin for total and cytoplasmic proteins, and histone H3 for nuclear proteins). OCLD: occludin; CLDN3: claudin-3; E-cad: E-cadherin.

The original full Western blots images are depicted as follows:

**Repeat A**

β-actin (1), 42 kD
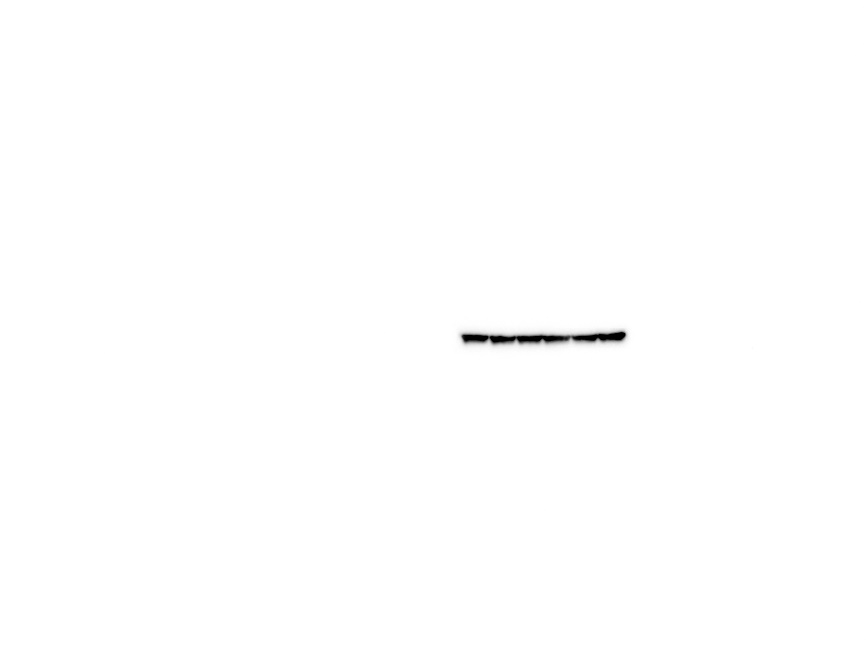


CLND3, 23kD
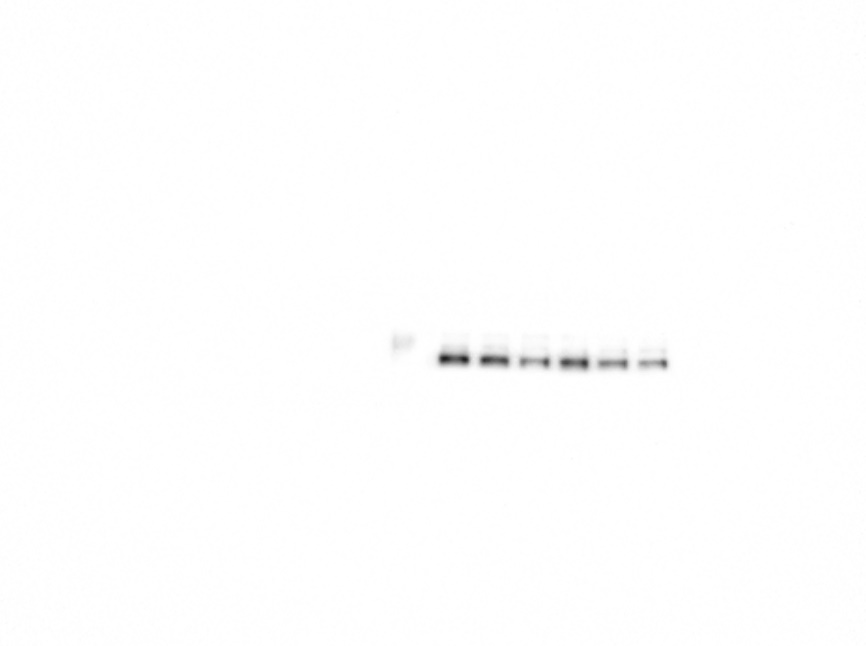


E-cad, 120 kD
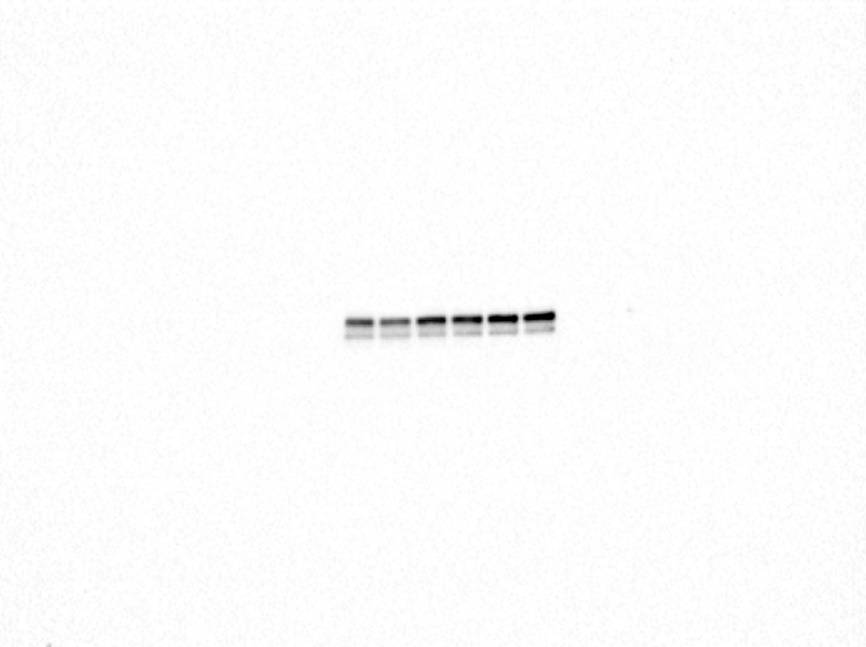


HIF-1α, 93 kD
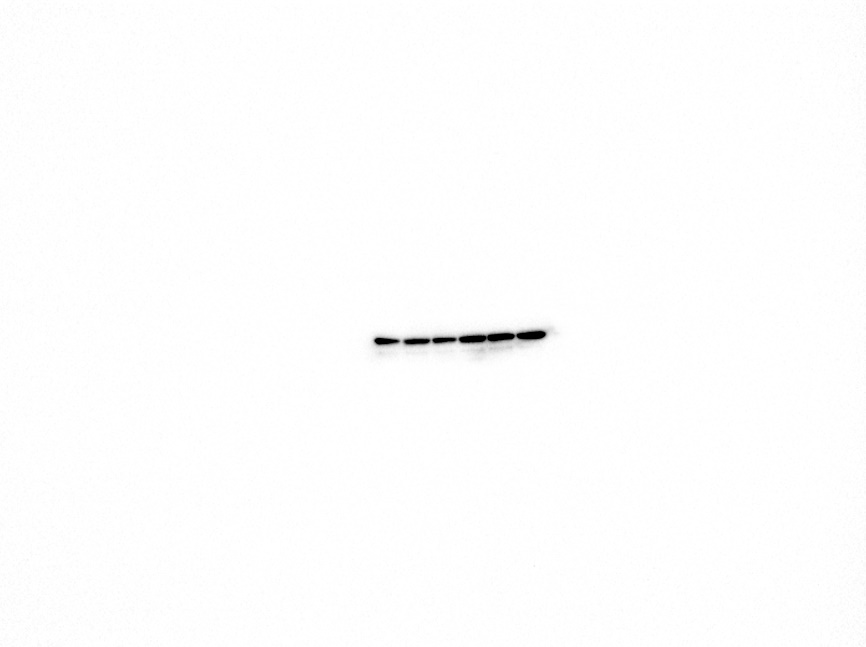


HSF-1, 80 kD
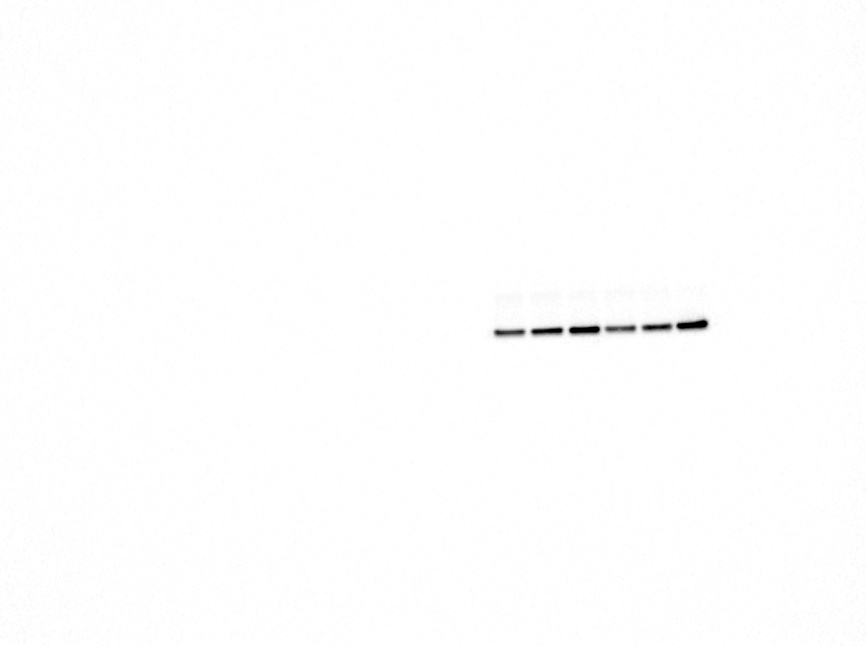


HSP-70, 70 kD
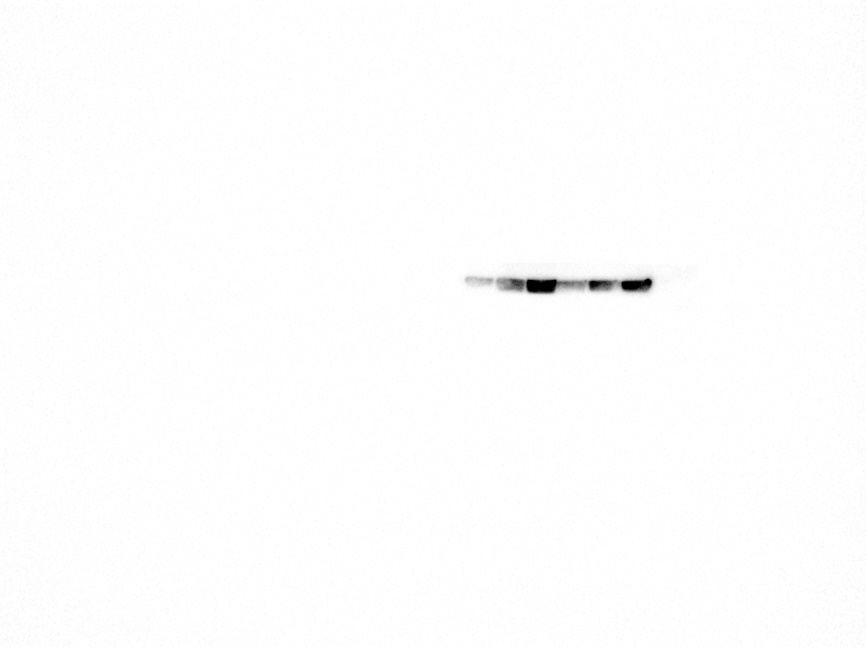


OCLD, 62 kD
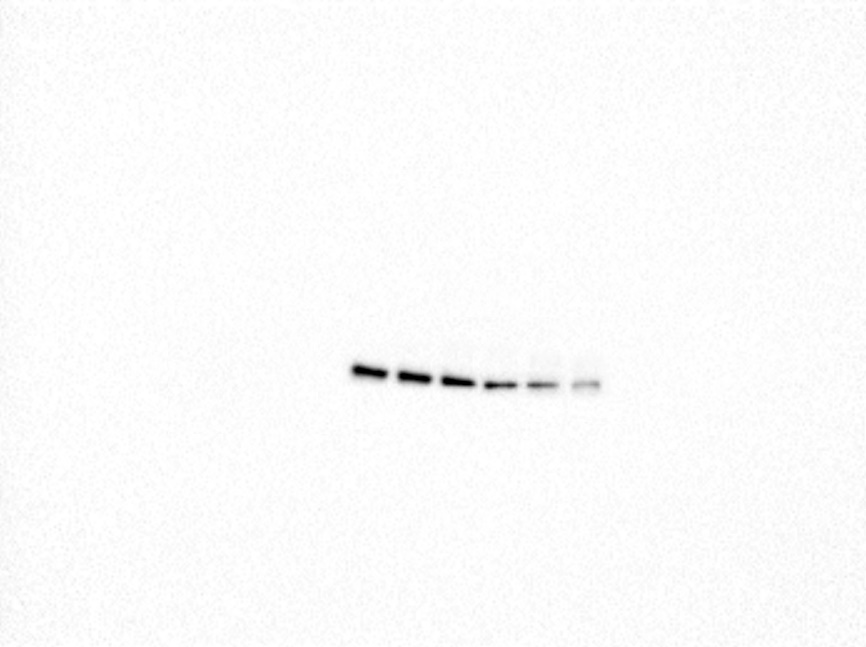


ZO-1, 255 kD
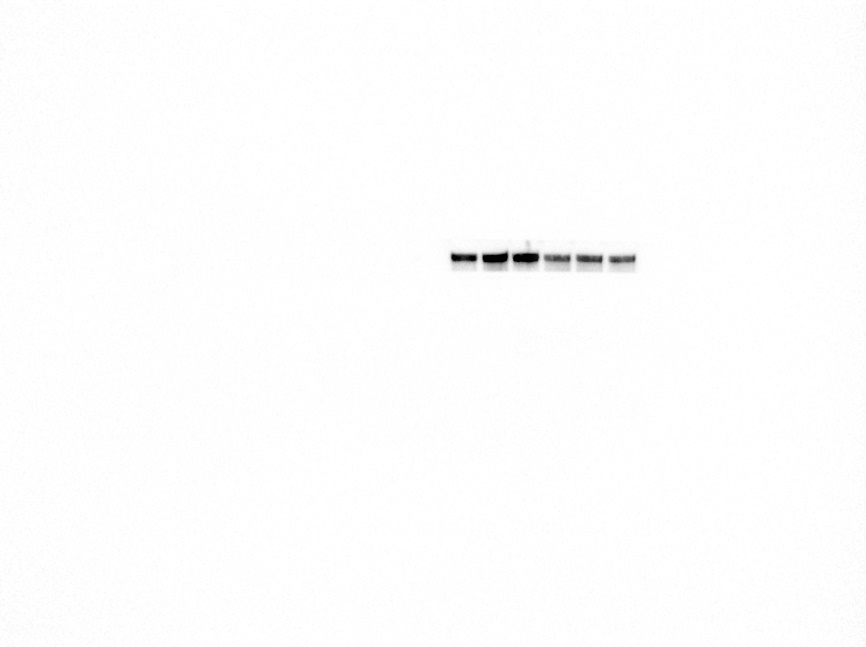


β-actin (2), 42 kD
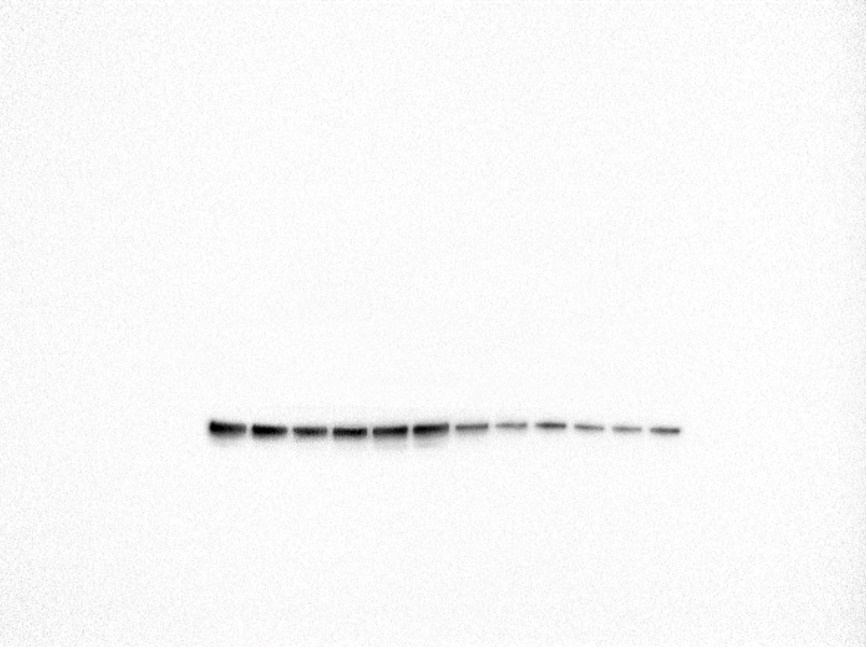


histone h3, 18 kD
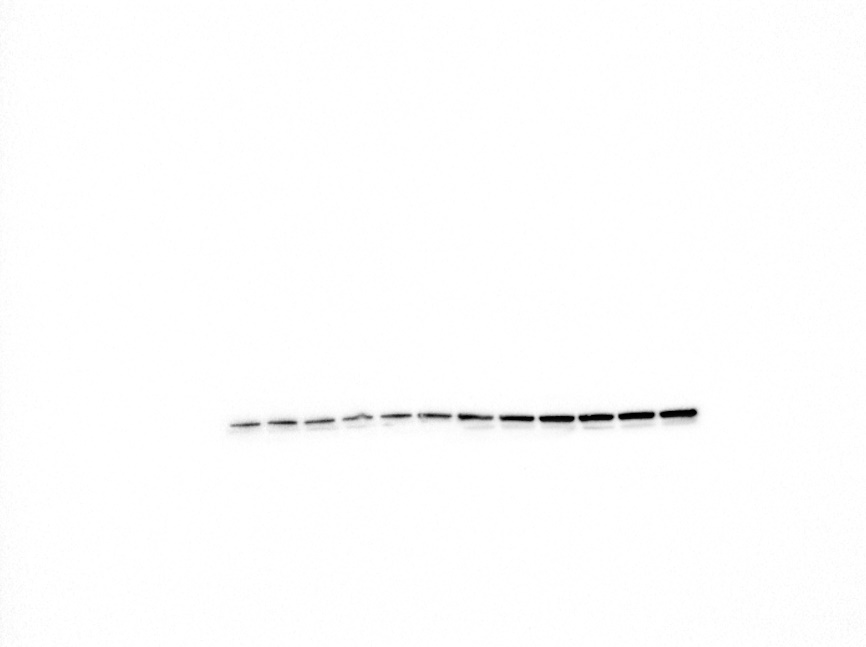


Nrf2, 98 kD
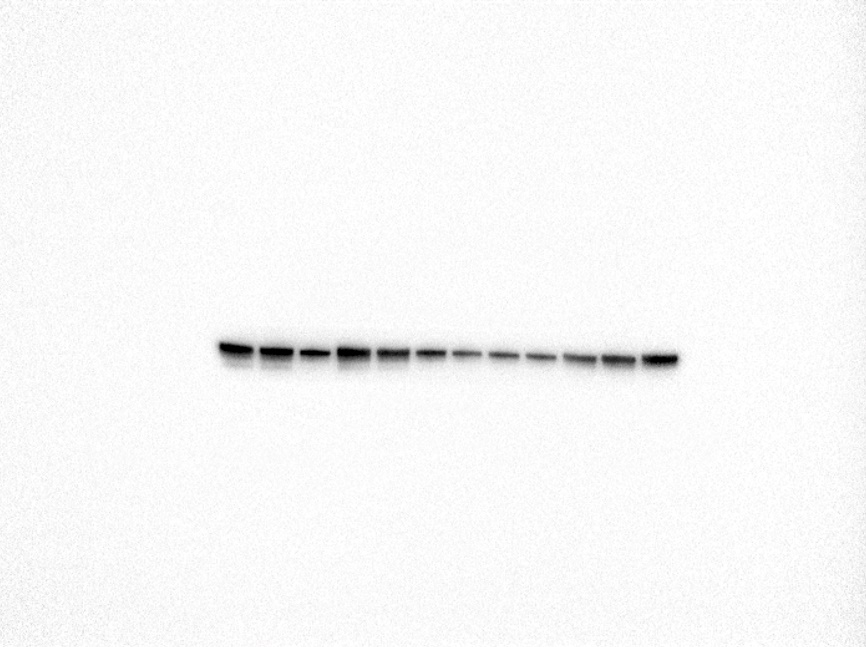


**Repeat B**

β-actin (1), 42 kD
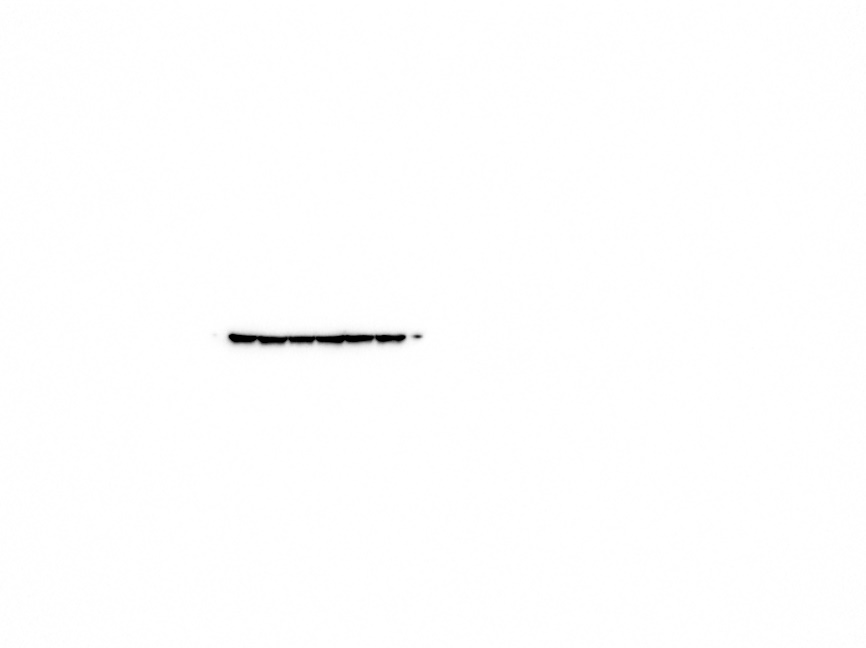


HSP-70, 70 kD
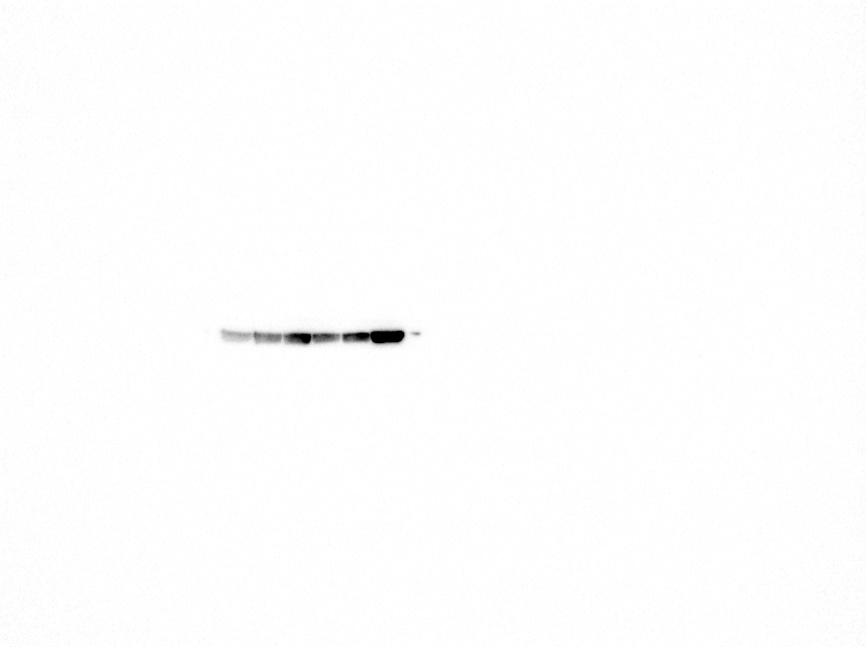


β-actin (2), 42 kD
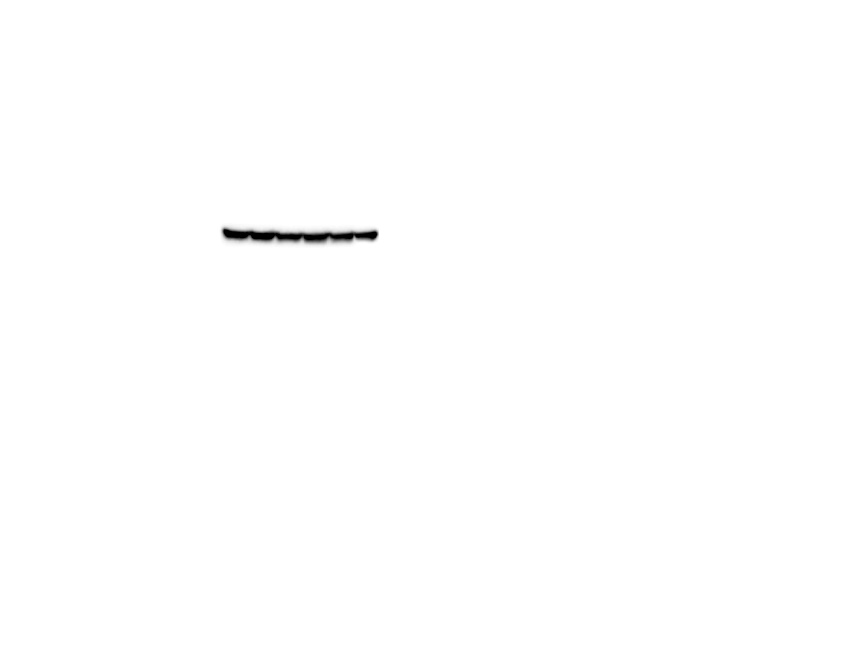


CLND3, 23 kD
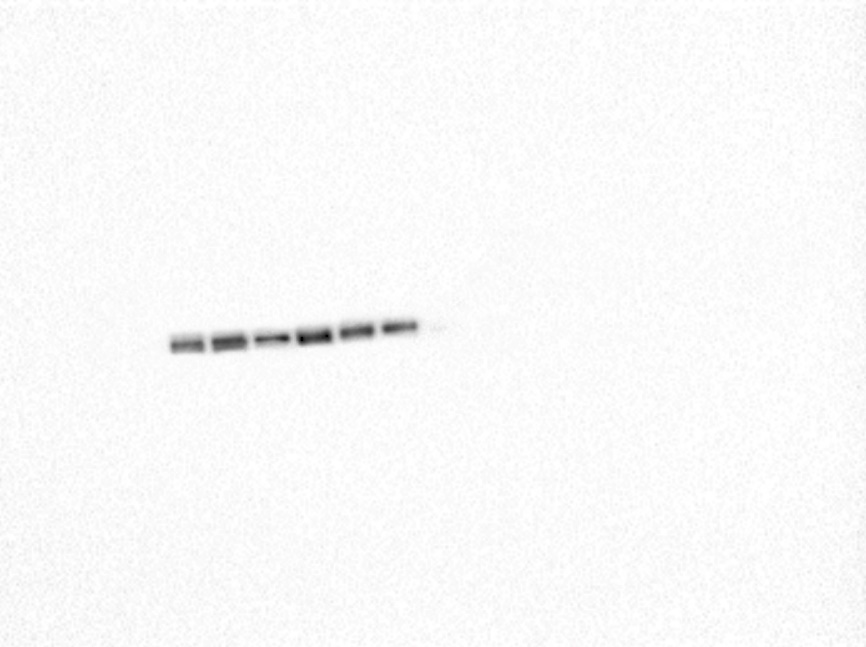


E-cad, 120 kD
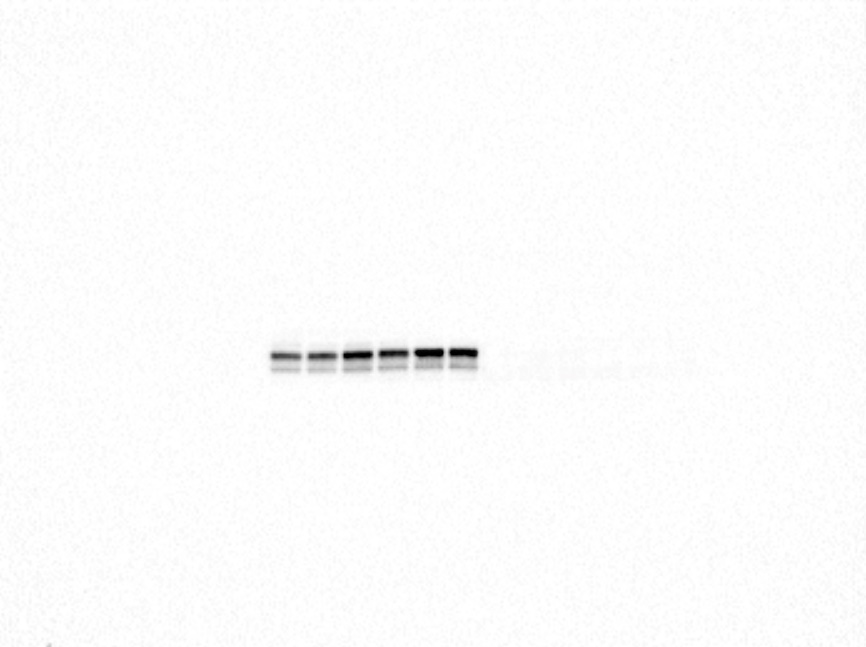


HIF-1α, 93 kD
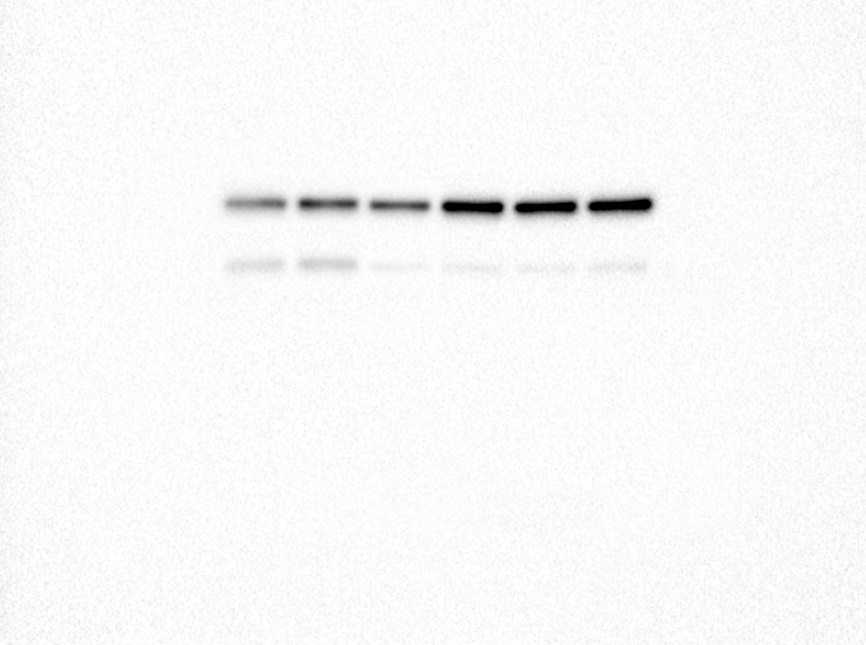


HSF-1, 80 kD
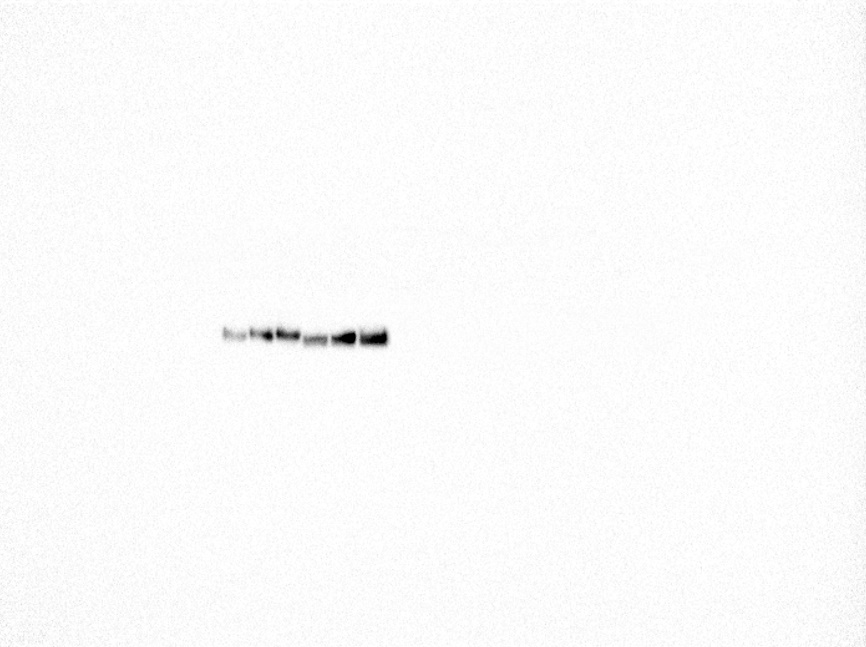


OCLD, 62 kD
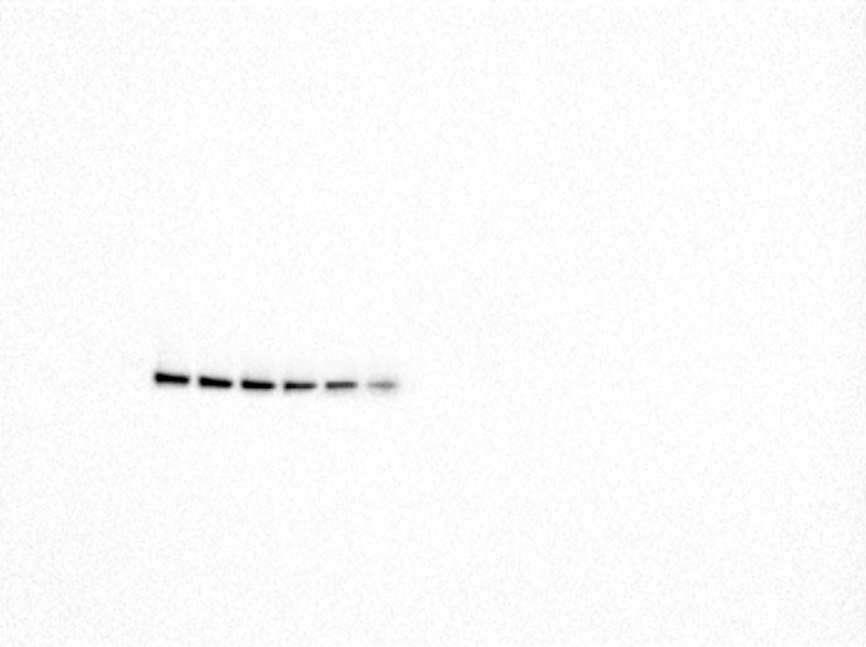


ZO-1, 255 kD
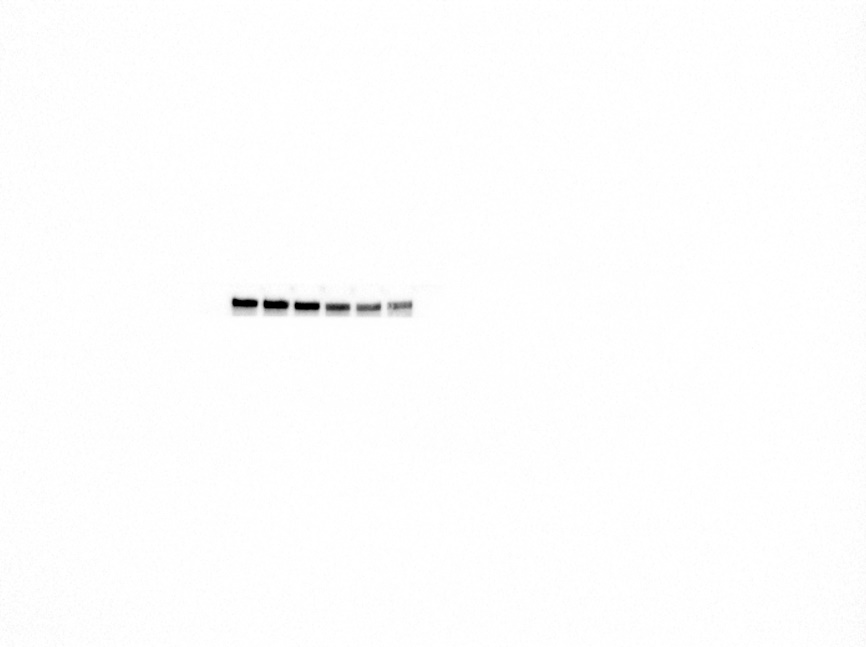


β-actin (3), 42 kD
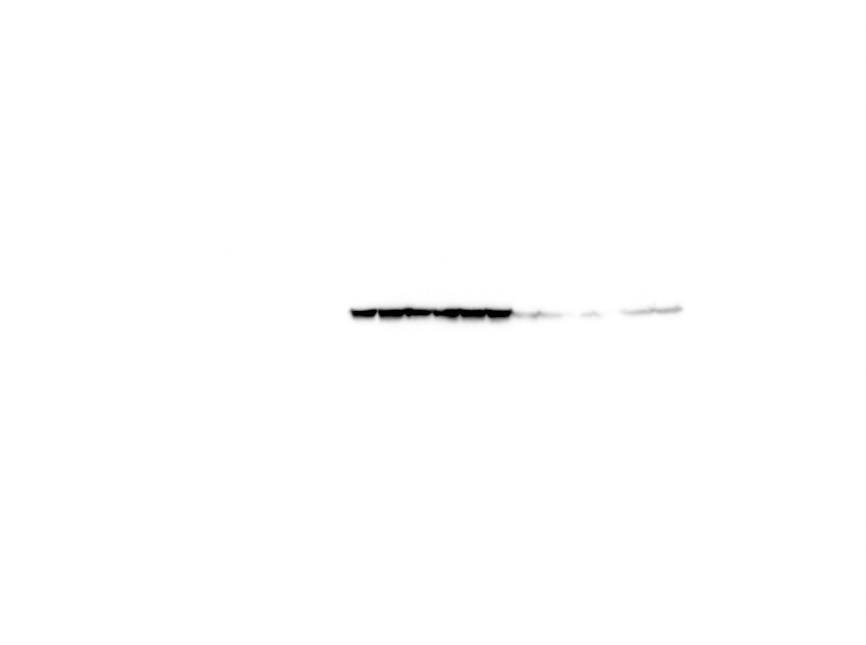


histone h3, 18 kD
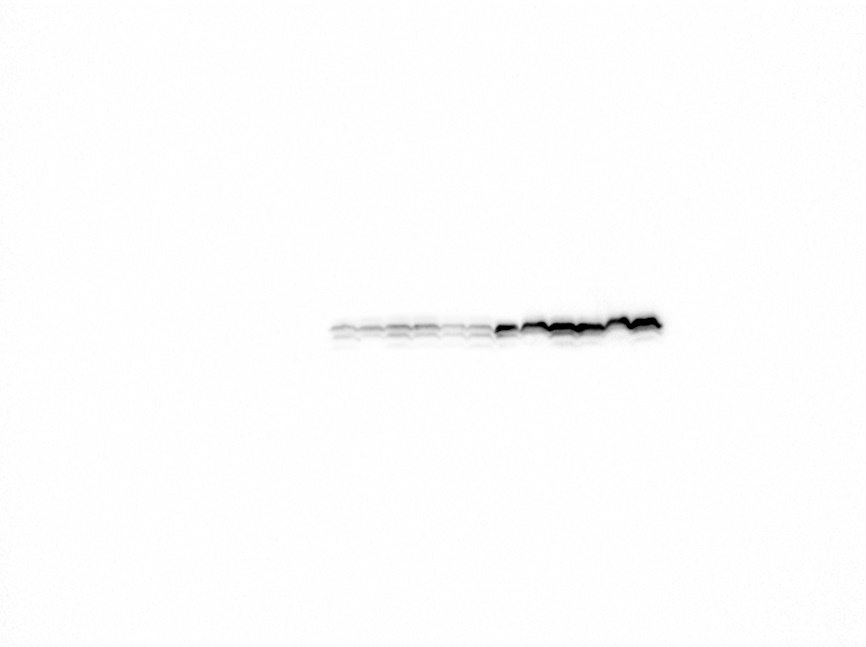


Nrf2, 98 kD
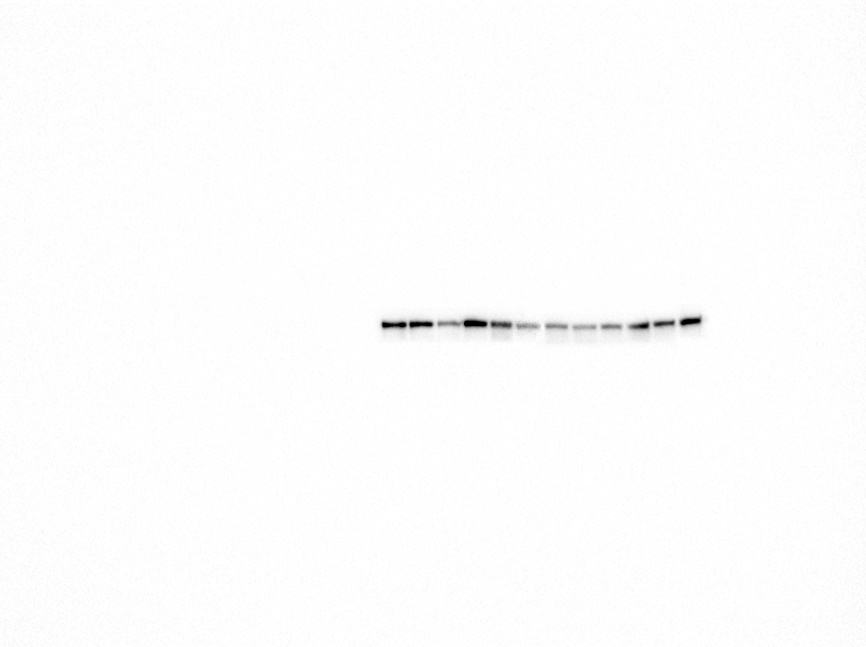


**Repeat C**

β-actin (1), 42 kD
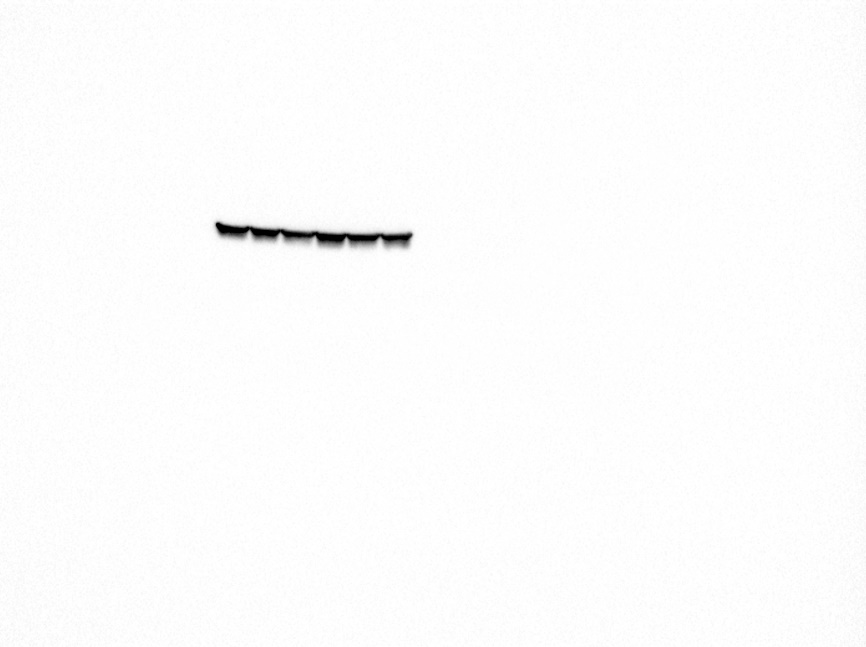


CLND3, 23 kD
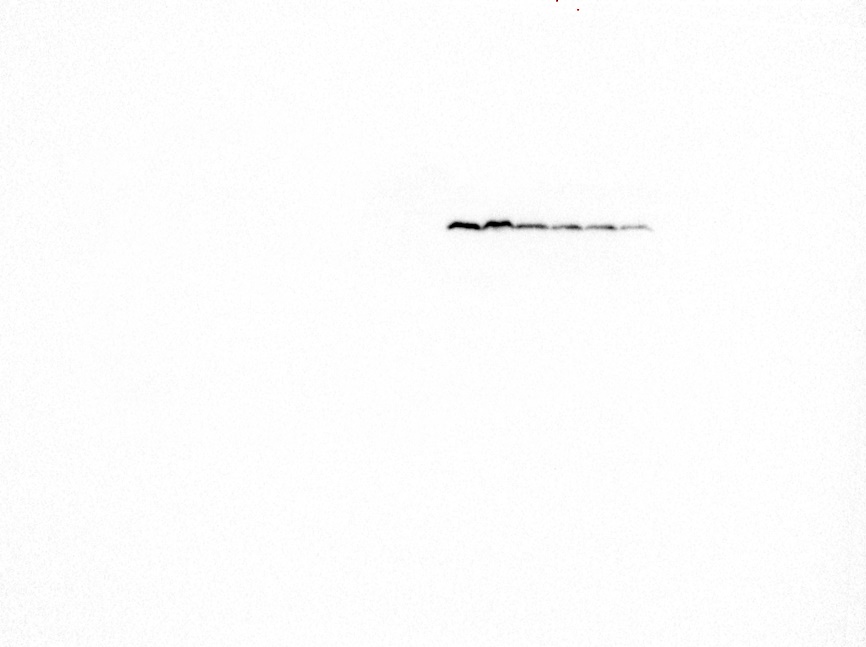


E-cad, 120 kD
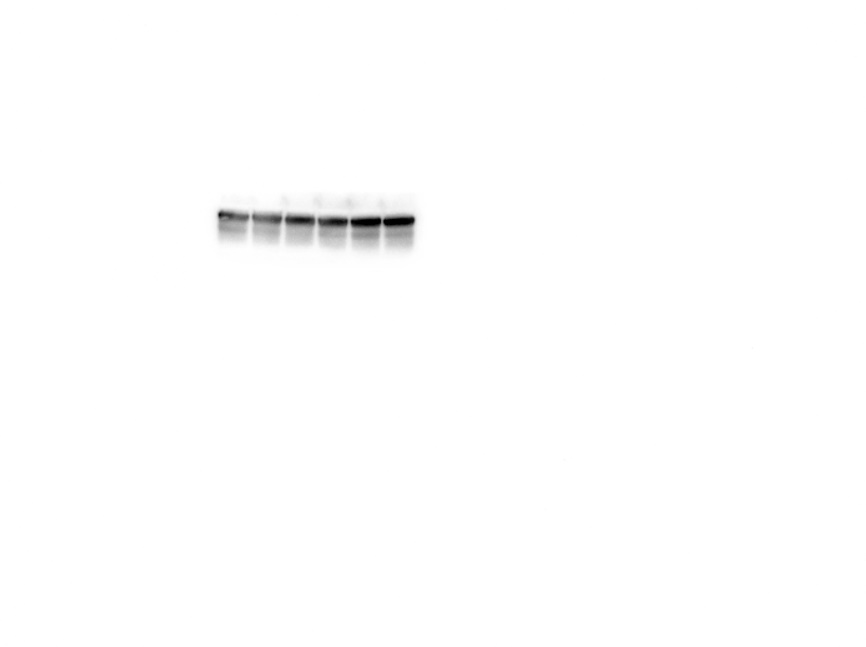


HIF-1α, 93 kD
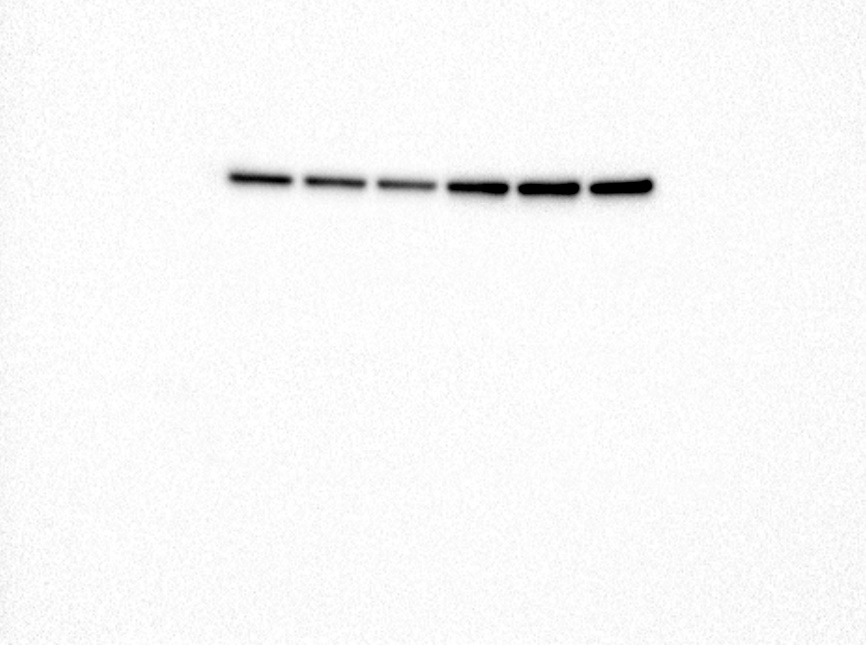


HSF-1, 80 kD
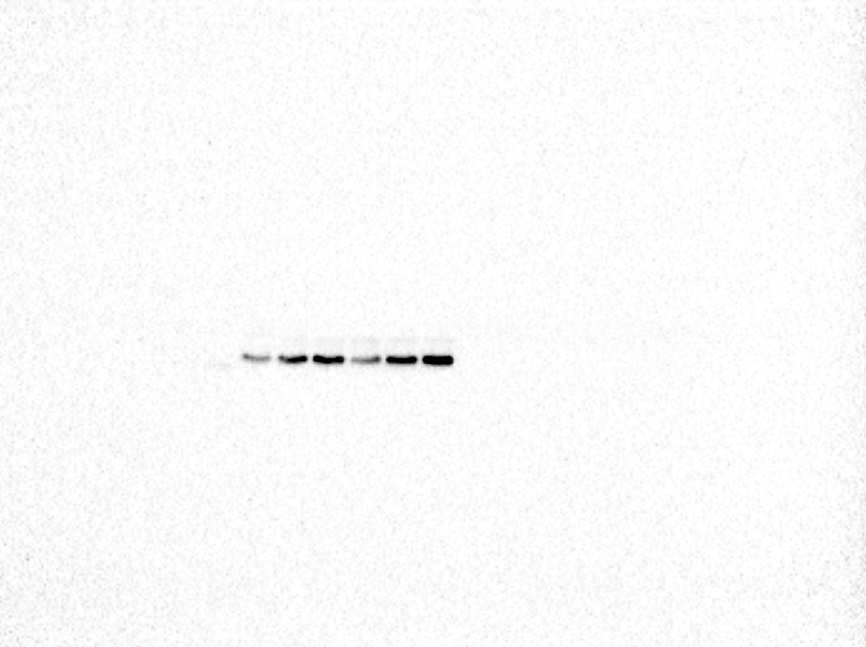


HSP-70, 70 kD
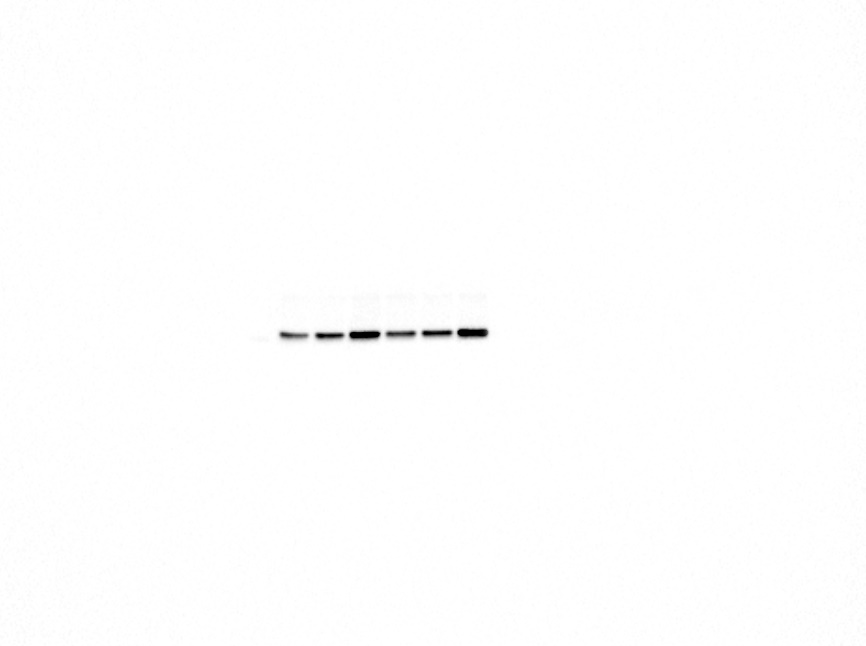


OCLD, 62 kD
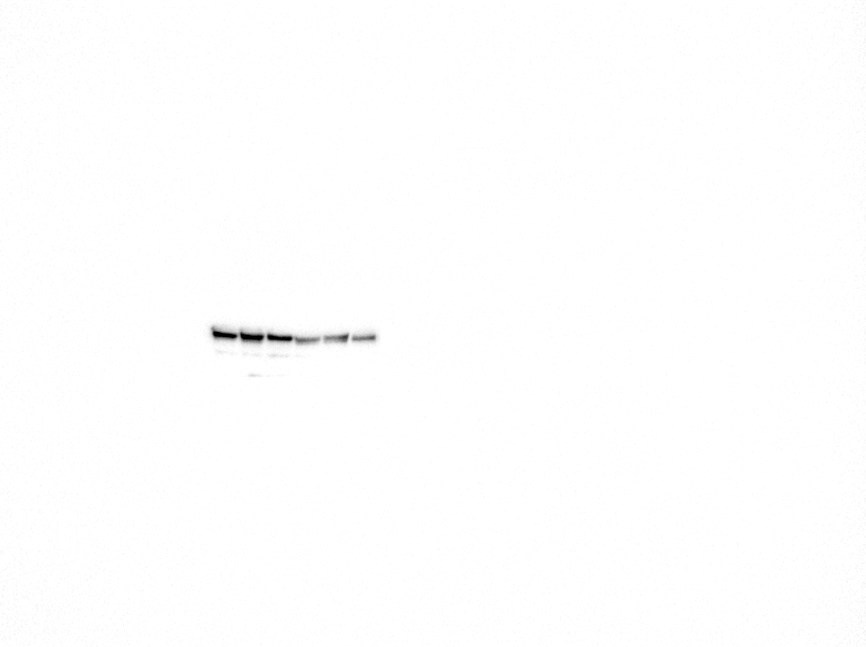


ZO-1, 225 kD
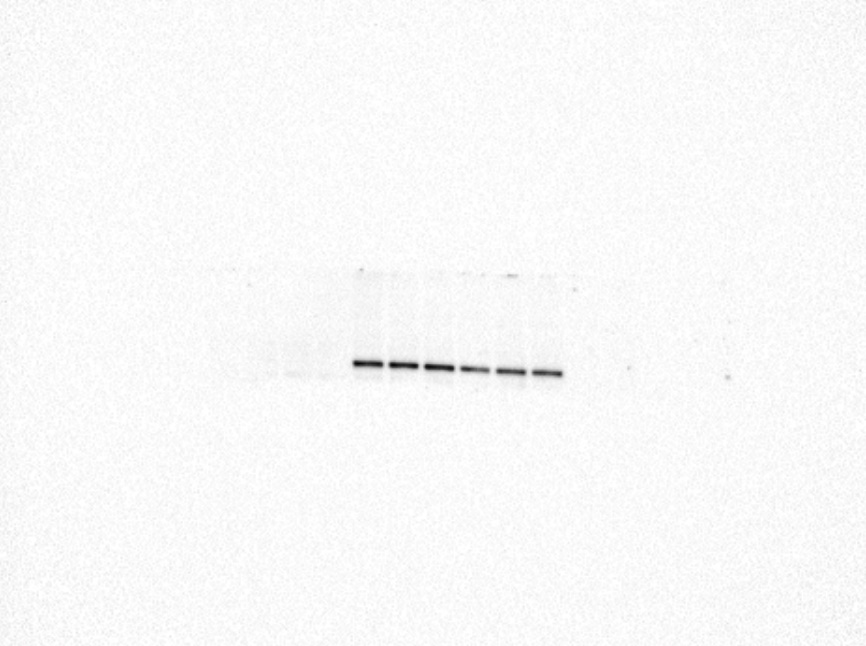


β-actin (2), 42 kD
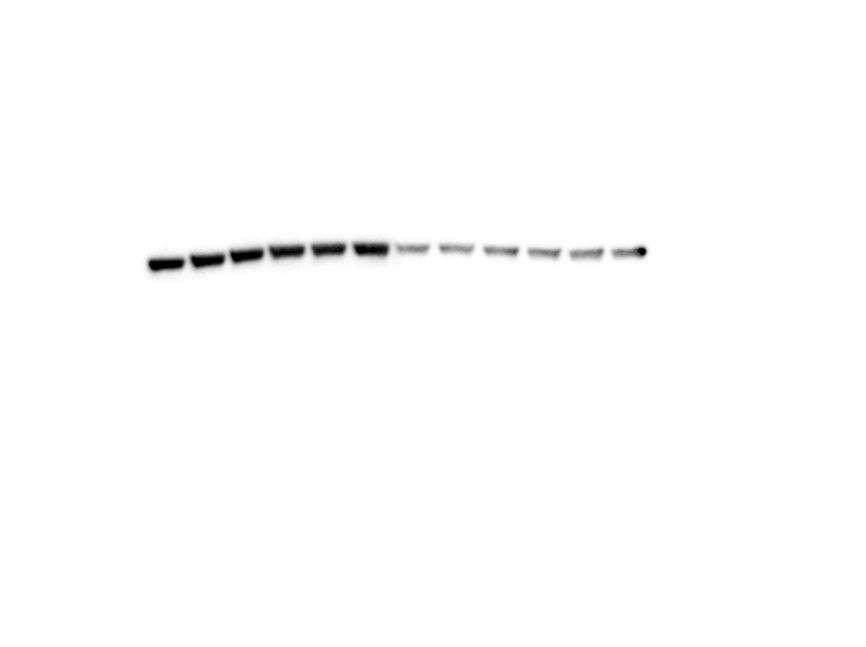


histone h3, 18 kD
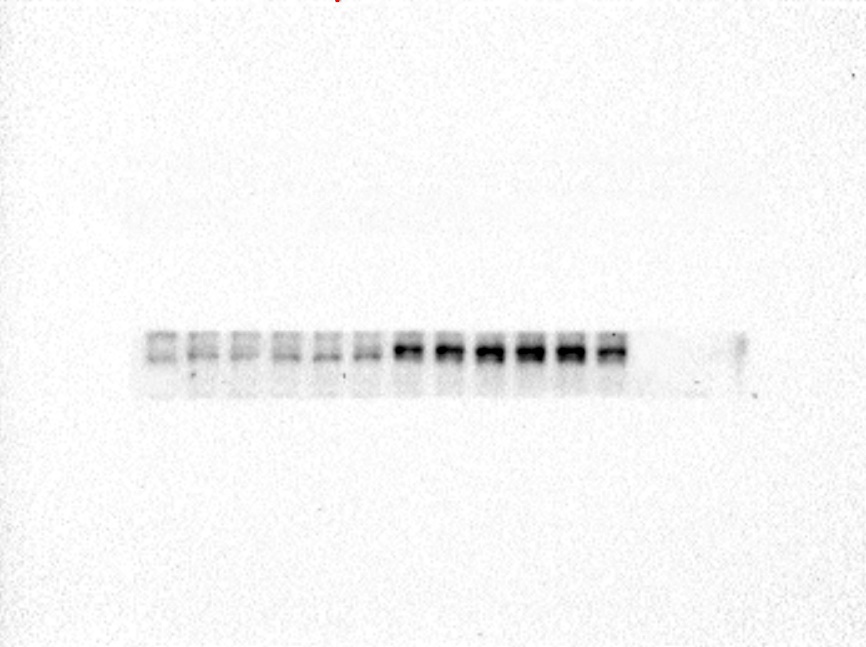


Nrf2, 98 kD
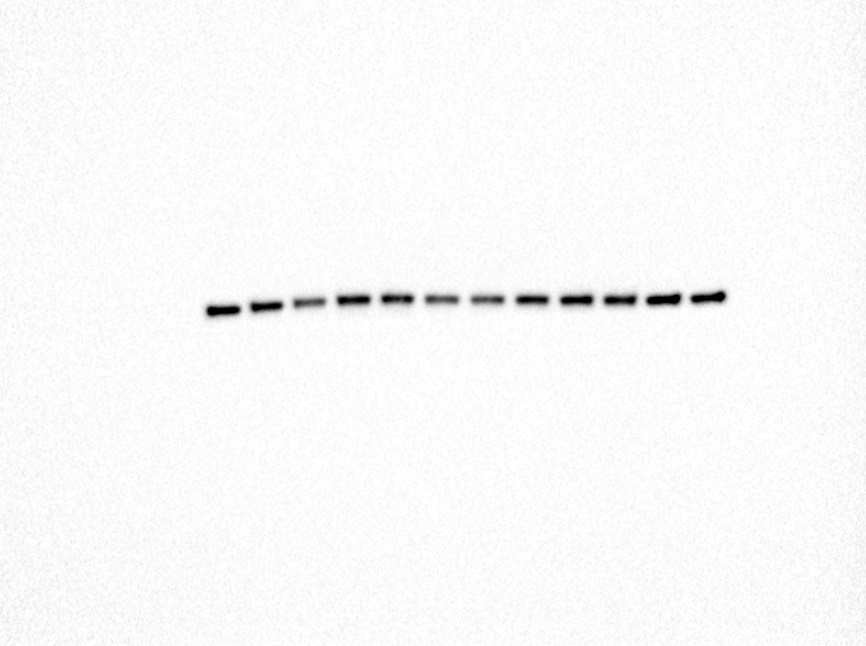

Supplement: Supplementary file 1 — Supplementary Information. [file 41598_2021_92574_MOESM1_ESM.docx]
